# The Potential of Usnic-Acid-Based Thiazolo-Thiophenes as Inhibitors of the Main Protease of SARS-CoV-2 Viruses

**DOI:** 10.3390/v16020215

**Published:** 2024-01-31

**Authors:** Olga I. Yarovaya, Aleksandr S. Filimonov, Dmitriy S. Baev, Sophia S. Borisevich, Anna V. Zaykovskaya, Varvara Yu. Chirkova, Mariya K. Marenina, Yulia V. Meshkova, Svetlana V. Belenkaya, Dmitriy N. Shcherbakov, Maxim A. Gureev, Olga A. Luzina, Oleg V. Pyankov, Nariman F. Salakhutdinov, Mikhail V. Khvostov

**Affiliations:** 1 Department of Medicinal Chemistry, N.N. Vorozhtsov Novosibirsk Institute of Organic Chemistry SB RAS, 630090 Novosibirsk, Russia; alfil@nioch.nsc.ru (A.S.F.); mitja2001@gmail.com (D.S.B.); mareninamk@nioch.nsc.ru (M.K.M.); meshkova_29@mail.ru (Y.V.M.); belenkaya.sveta@gmail.com (S.V.B.); dnshcherbakov@gmail.com (D.N.S.); luzina@nioch.nsc.ru (O.A.L.); anvar@nioch.nsc.ru (N.F.S.); khvostov@nioch.nsc.ru (M.V.K.); 2 Synchrotron Radiation Facility SKIF, G.K. Boreskov Institute of Catalysis SB RAS, 630559 Koltsovo, Russia; monrel@mail.ru; 3 Laboratory of Chemical Physics, Ufa Institute of Chemistry, Ufa Federal Research Centre, 450078 Ufa, Russia; 4 State Research Center of Virology and Biotechnology VECTOR, Rospotrebnadzor, 630559 Koltsovo, Russia; zaykovskaya_av@vector.nsc.ru (A.V.Z.); pyankov_ov@vector.nsc.ru (O.V.P.); 5 Institute of Biology and Biotechnology, Altay State University, 656049 Barnaul, Russia; varvara.chirkova@gmail.com; 6 Laboratory of Bio- and Cheminformatics, St. Petersburg School of Physics, Mathematics and Computer Science, HSE University, 194100 St. Peterburg, Russia; max_technik@mail.ru

**Keywords:** usnic acid, SARS-CoV-2, main protease, 3CL^pro^, molecular modeling

## Abstract

Although the COVID-19 pandemic caused by SARS-CoV-2 viruses is officially over, the search for new effective agents with activity against a wide range of coronaviruses is still an important task for medical chemists and virologists. We synthesized a series of thiazolo-thiophenes based on (+)- and (−)-usnic acid and studied their ability to inhibit the main protease of SARS-CoV-2. Substances containing unsubstituted thiophene groups or methyl- or bromo-substituted thiophene moieties showed moderate activity. Derivatives containing nitro substituents in the thiophene heterocycle—just as pure (+)- and (−)-usnic acids—showed no anti-3CL^pro^ activity. Kinetic parameters of the most active compound, **(+)-3e**, were investigated, and molecular modeling of the possible interaction of the new thiazolo-thiophenes with the active site of the main protease was carried out. We evaluated the binding energies of the ligand and protein in a ligand–protein complex. Active compound **(+)-3e** was found to bind with minimum free energy; the binding of inactive compound **(+)-3g** is characterized by higher values of minimum free energy; the positioning of pure (+)-usnic acid proved to be unstable and is accompanied by the formation of intermolecular contacts with many amino acids of the catalytic binding site. Thus, the molecular dynamics results were consistent with the experimental data. In an in vitro antiviral assay against six strains (Wuhan, Delta, and four Omicron sublineages) of SARS-CoV-2, **(+)-3e** demonstrated pronounced antiviral activity against all the strains.

## 1. Introduction

In May 2023, the WHO declared the end of the COVID-19 pandemic caused by the novel coronavirus SARS-CoV-2. Nonetheless, this does not mean that the threat of exposure to the virus has disappeared, nor does it mean that the likelihood of severe, even fatal, disease is zero [1]. The virus continues to circulate in the human population, and its new mutants that are resistant to existing vaccines can theoretically lead to a new outbreak. Reduced rates of vaccination against SARS-CoV-2 have also contributed to an increase in disease incidence as the global epidemiological situation has improved and most countries have returned to their normal activities. In addition, current strains of the virus can be dangerous for the elderly and people with various comorbid conditions, increasing mortality in the population and stress on the healthcare system [2]. Therefore, it is important to develop effective pharmacotherapies that can reduce the severity of the disease and the frequency of hospital admissions.

The following proteins and processes are usually considered the main targets for the development of new antiviral medicines for COVID-19: helicases, transmembrane serine protease 2, cathepsin L, cyclin G-associated kinase, adaptor-associated kinase 1, two-pore channel, viral virulence factors, 3-chymotrypsin-like protease (3CL^pro^), papain-like protease [3], RNA-dependent RNA polymerase (RdRp) [4,5], an excessive inflammatory response, endocytosis, and viral membrane, nucleocapsid, envelope, and accessory proteins [6]. Despite this diversity of potential targets, only two of them have been successfully applied to drug design: RdRp and 3CL^pro^ [3]. 3CL^pro^ (also known as M^pro^ and nsp5) is responsible for the generation of 13 nonstructural proteins by cleaving pp1a and pp1ab (the main polyproteins encoded by the RNA genome of SARS-CoV-2), including RdRp, helicase, exoribonucleases, 2′-O-methyltransferase, and uridine-specific endoribonuclease [7,8].

Apparently, the key factor in the inhibitory effect on M^pro^ may be the ability of an inhibitor to bind amino acid residues of the flexible loop region (amino acid residues 141–145), whose Gly143, Ser144, and Cys145 form the oxyanion hole of M^pro^ [9]. The oxyanion hole is a pocket (in the active site of an enzyme) that stabilizes a transition state’s negative charge on the deprotonated oxygen, or alkoxide. Stabilization of the transition state reduces the activation energy required for the reaction and thus promotes catalysis [10]. The catalytic center of the enzyme in question consists of two pockets: S1′, where interaction with His41 occurs, and S2′, where the catalytic amino acid residue Cys145 is located. Additionally, research shows that the active site of M^pro^ is complemented by pockets that help stabilize the inhibitor near the catalytic center of the enzyme. In the S1 pocket, inhibitors can interact with amino acid residues Glu166 and Phe140; in the S2 pocket, with His41, Hie163, and His164; in the S3 pocket, with Gln189 and Met49; and in the S4 pocket, with Pro168 [9,11,12].

This is the mechanism of action of the drugs that have received approvals for COVID-19 pharmacotherapy. The first one is nirmatrelvir, which, together with ritonavir, is a component of Pfizer’s Paxlovid^®^ (Groton, CT, USA), approved by the FDA in 2023 for managing mild-to-moderate COVID-19 in adults who are at high risk of severe disease [13]. The second is ensitrelvir (brand name Xocova), discovered by Hokkaido University and Shionogi & Co., Ltd. (Osaka, Japan); this drug is in phase III trials but has already gotten emergency regulatory approval in Japan for clinical use [14]. In in vitro and in vivo tests, both have shown similar antiviral effects [15]. Thus, the search for new 3CL^pro^ inhibitors appears to be a promising area of research to expand the range of therapeutic agents against SARS-CoV-2 [16].

One of the encouraging fields of medicinal chemistry for the synthesis of new agents with antiviral activity is the use of natural compounds as a starting platform, which has recently received special attention [17,18,19,20]. We have earlier identified effective borneol-based inhibitors of entry of a wide range of SARS-CoV-2 viruses using a pseudoviral system [21], using a surrogate system of triterpene acid–based amides as inhibitors of the main viral protease [22]. Glycyrrhizic acid nicotinates have also been shown to be inhibitors of SARS-CoV-2 viruses [23]. In our search for new effective inhibitors of SARS-CoV-2, we have studied the properties of (+)-usnic acid and its derivatives. Previously, it has been demonstrated that usnic acid is active against different coronavirus strains [24]. We recently found that thiazohydrazones based on (+)- and (−)-usnic acid containing furan moieties, pyrrole moieties, or indole moieties, as well as p-methoxybenzylidene derivatives with various substituents at the *m*-position, possess activity against the main viral protease [25]. Compounds containing a furan moiety have demonstrated the highest activity against both proteases and infectious viruses. At the same time, it is well known that furan derivatives can often be unstable both during storage and in biological fluids as well as in living organisms.

Thiophene has earned the sobriquet of the “wonder heterocycle” due to a wide range of biological activities, such as anticancer [26], antimicrobial [27], anti-inflammatory [28], antidepressant [29], analgesic [30], and anticonvulsant [31]. The biological effects of thiophene compounds may be related to their metabolism in living organisms, and it has been suggested that substituted and condensed thiophenes are less toxic [32]. Thus, the investigation of the antiviral activity of compounds containing a thiophene group attracts considerable attention from researchers. In particular, substituted imidazobenzothiazoles containing bromothiophene have demonstrated high activity against the influenza virus [33]. Recently, Zika virus inhibitors containing different substituents in the thiophene moiety were described, and it was shown that they are non-nucleoside inhibitors of Zika virus RdRp [34]. 2-Nitro-substituted thiophenes have demonstrated a broad spectrum of activity against influenza viruses and upregulate key antiviral interferon-stimulating genes *MxA*, *OAS2*, and *CH25H* [35]. According to the analysis of literature data, in the present study, we synthesized both (+)- and (−)-usnic-acid derivatives containing unsubstituted thiophenes or methyl-, bromo-, or nitrothiophenes and tested in silico and in vitro their activity against the main viral protease of SARS-CoV-2.

## 2. Materials and Methods

### 2.1. Chemistry

Analytical and spectral experiments were performed at the Multi-Access Chemical Service Center of the Siberian Branch of the Russian Academy of Sciences.

^1^H- and ^13^C-NMR spectra of solutions of compounds in CDCl_3_ were recorded by means of a Bruker AV-400 spectrometer (at 400.13 and 100.61 MHz, respectively). Residual signals of the solvent were used as references (δ_H_ 7.24 and δ_C_ 76.90 for CDCl_3_). Mass spectra (70 eV) were recorded on a DFS Thermo Scientific high-resolution mass spectrometer (Waltham, MA, USA). Melting points were measured using a Kofler heating stage. Specific rotation was determined by means of PolAAr 3005 (Optical Activity Ltd., Huntingdon, UK) and is provided in (deg × mL) × (g × dm)^−1^ units, whereas the concentration of solutions is shown in g × (100 × mL)^−1^. Merck silica gel (63–200 µ) was used for column chromatography. Thin-layer chromatography was performed on TLC silica gel 60F_254_ (Merck KGaA, Darmstadt, Germany).

(R)-(+)-Usnic acid **(+)-1** was acquired from Zhejiang Yixin Pharmaceutical Co., Ltd. (Jinhua, China). Synthetic starting materials, reagents, and solvents were purchased from Sigma-Aldrich (St. Louis, MO, USA), Acros Organics, (Geel, Belgium) and AlfaAesar (Haverhill, MA, USA) (95–99% pure). All chemicals were used as described, unless stated otherwise. Reagent-grade solvents were redistilled prior to use.

(S)-(−)-Usnic acid **(−)-1** (α_D_ −456 [c 0.1, CHCl_3_]) was isolated from *Cladonia stellaris* by a procedure from ref. [36]. Bromousnic acids **(+)-2** and **(−)-2** were synthesized according to the literature [37].

#### 2.1.1. The General Procedure for the Synthesis of Compounds **5a–g**

One mmol of a corresponding aldehyde (**4a–g**) was dissolved in 2 mL of ethyl alcohol. The resulting solution was slowly added dropwise, with stirring, to a solution of 1.1 mmol of thiosemicarbazide in 2 mL of distilled water. The precipitate that formed was filtered off, washed with water, and then air dried. Obtained thiosemicarbazones **5a–g** were isolated in 76–93% yields. The spectra of the substances matched the published ones [37].

#### 2.1.2. The General Procedure for the Synthesis of Compounds **3a–g**

A mixture of compound **(+)-2** or **(−)-2** (1 mmol, 423 mg) and a corresponding thiosemicarbazone (**5a–g**, 1 mmol) was heated under reflux in 25 mL of MeOH for 0.5–2.0 h. The reaction mixture was cooled and poured into 75 mL of water. After that, the reaction mixture was cooled; the precipitate that formed was filtered off, washed with MeOH, and air dried. The precipitate was placed in a separatory funnel, 30 mL of methylene chloride was added, a suspension was created, and 20 mL of a saturated solution of NaHCO_3_ was introduced. The mixture was shaken vigorously several times until the suspension completely turned into a dark red solution. This solution was separated from the aqueous layer, washed with 20 mL of water once, and evaporated in a rotary evaporator. Obtained compounds **3a–g** were isolated in 65–78% yields. The spectra of the substances matched the published ones [37]. 

### 2.2. Biological Experiments

#### 2.2.1. Enzyme Inhibition Experiments

The preparation of the main SARS-CoV-2 protease, 3CL^pro^, was carried out in a previously obtained transformant *E. coli* strain, which ensures the synthesis of the target protein in soluble form. The standard cultivation protocol included the addition of the inducer IPTG. The purification of 3CL^pro^ included cell biomass lysis and ultrasonic disintegration, as well as the purification of the clarified lysate on Ni-Sepharose [38]. The purity of the resulting sample of the main coronavirus protease, 3CL^pro^, was assessed by SDS-PAGE under denaturing conditions according to the Laemmli method. Protein concentration was determined by the Bradford assay [39].

To determine the half-maximal inhibitory concentration of the test compounds and the kinetic parameters of the main coronavirus protease (with or without potential inhibitory compounds), fluorescence was measured in the kinetic scanning mode.

Fluorescence was observed by an assay involving a synthetic fluorescently labeled peptide substrate of the type DabcylKTSAVLQ↓SGFRKME(Edans)NH_2_ (more than 95% purity, CPC Scientific Inc., Hangzhou, China) containing the site digested by the main protease of coronavirus. The fluorescent dye attached to the peptide substrate allows the FRET effect to be implemented: the interaction of recombinant 3CL^pro^ of SARS-CoV-2 with the peptide substrate cleaves the above site, thereby leading to an increase in fluorescence intensity due to the physical distancing of the fluorophore from the quencher. The level of fluorescence resulting from peptide substrate cleavage by the main protease 3CL^pro^ was recorded on a CLARIOstar Plus microplate fluorimeter-spectrophotometer (BMG Labtech, Ortenberg, Germany) and SuPerMax 3100 (Flash Spectrum, Shanghai, China) at 355 and 460 nm for excitation/emission, respectively. Reaction mixtures contained Tris-HCl buffer (pH 7.3) supplemented with ethylenediaminetetraacetic acid (EDTA) and dithiothreitol (DTT), a synthetic peptide substrate, recombinant 3CL^pro^ at 300 nM, and an inhibitory compound. When IC_50_ was being determined, the concentration of the substrate in the well was 10 µM, and the concentration of the inhibitor was varied from 400 to 0 µM. During the determination of kinetic parameters, the concentration of the inhibitor was equal to its IC_50_ measured earlier, and the concentration of the substrate was varied in the range of 10 to 0 µM.

The instrument was calibrated using a cell containing the peptide subjected to complete hydrolysis; this fluorescence level was set to 80%. The measurements were carried out in triplicate in the kinetic scanning mode.

The rate of the enzymatic reaction was determined from the kinetic fluorescent curves via calculation of the change in fluorescence per unit time at different concentrations of the substrate. The accompanying MARS data analysis software 4.2 (BMG Labtech, Germany) were employed to calculate IC_50_, K_m_, and V_max_. Based on the obtained values of K_m_ and V_max_, the number of enzyme turnovers (k_cat_ = V_max_/[E]) and the enzyme’s catalytic efficiency (k_cat_/K_m_) were computed, which allowed us to evaluate the effectiveness of suppression of the kinetic reaction by a potential inhibitor.

#### 2.2.2. Evaluation of the Antiviral Activities against SARS-CoV-2 Viruses

The SARS-CoV-2 in vitro experiment was conducted in laboratories at Biosafety Level 3 (BSL-3). It was carried out using the following six coronavirus strains, which belong to different genetic lines: B.1.1, B.1.617.2, BA.1, BA.5.2, BQ.1.1, and XBB.1.5 (the State Collection of Pathogens of Viral Infections and Rickettsioses at the State Research Center of Virology and Biotechnology VECTOR, Rospotrebnadzor, Russia).

The viruses were propagated in cultured Vero E6 cells. These cells were grown in 96-well culture plates to a confluence of at least 95%. Samples of compounds were dissolved in dimethyl sulfoxide (DMSO) to a concentration of 10 mg/mL. Remdesivir served as a control drug. Half-maximal effective concentrations (EC_50_) of compounds were evaluated in an assay of reduction of the cytopathic effect on cells. Serial three-fold dilutions of the compounds were prepared, starting at 600 μg/mL. To test each compound, virus doses of 100 TCID_50_ (50% tissue culture infectious doses) per well were applied.

The inhibitory activities and toxicity of the tested compounds were assessed simultaneously. Specifically, dilutions of the compounds were added into the wells in the culture plates containing a monolayer of the cells. A plain medium (to determine the toxic concentration of the tested compounds) or a medium containing a virus (to determine inhibitory activities) was then added. The culture plates were incubated at 37 °C for 4 days. An MTT (3-(4,5-dimethylthiazol-2-yl)-2,5-diphenyltetrazolium bromide) (NeoFroxx) 0.5 mg/mL solution was prepared in sterile phosphate-buffered saline, and 100 μL was then added into each well and incubated at 37 °C for 4 h. Next, the supernatant from each well was carefully removed by aspiration without disturbing the cells. Then, 100 μL of DMSO was added to each well to dissolve formazan crystals. The optical density was measured on a microplate reader (ThermoScientific Multiskan FC) at 570 nm. Data processing were carried out in the SOFTmax PRO 4.0 software by a 4-parameter method. Half-maximal cytotoxic concentration (CC_50_) and half-maximal effective concentration (EC_50_) were both determined.

### 2.3. Molecular Modeling

All theoretical calculations were performed using Schrodinger Small Molecule Drug Discovery Suite 2022-1 software [40].

#### 2.3.1. Protein and Ligand Preparation

Geometric parameters of proteins were downloaded from the noncommercial database Protein Data Bank [41]. A SARS-CoV-2 main protease M^pro^ XRD model (7L0D [42]) was chosen for molecular modeling. The model protein structure was prepared using Schrodinger Protein Prepwizard tools: hydrogen atoms were added and minimized, missing amino acid side chains were added, bond multiplicity was restored, solvent molecules were removed, and the entire structure was optimized in the OPLS4 force field [43]. 

The geometric parameters of the ligands were optimized by the force field method using the OPLS4 force field, considering all possible conformations. 

#### 2.3.2. Analysis of a Potential Binding Site 

SARS-CoV-2 M^pro^ is a functional homodimer that contains two identical active sites. For calculations, we used only one subunit of M^pro^, its monomer, which carries one active site, containing catalytic amino acids Cys145 and His41 and located in a cleft between two N-terminal domains: I and II [12].

#### 2.3.3. The Molecular Docking Procedure

Molecular docking was performed via a forced ligand positioning protocol (Glide induced fit docking, or Glide IFD) under the following conditions: flexible protein and ligand, 20 Å grid matrix size, and amino acids within 5 Å of the ligand were constrained to be optimized for a ligand influence. Docking solutions were ranked by evaluation of the following calculation parameters: the docking score (based on GlideScore with exclusion of penalties), ligand efficiency (LE, where the per-heavy-atom distribution of the scoring function is considered), and the model energy value parameter (Emodel), including GlideScore, energy of unbound interactions, and parameters of energy expended on the formation of compound stacking in the binding site.

#### 2.3.4. Molecular Dynamics Simulations

Ligand-protein complexes were placed in a virtual cube with a 15 Å buffer filled with a 0.15 M NaCl solution. The aqueous solvent model TIP3P was chosen. The NPT ensemble was utilized to simulate the system at a temperature of 310 K and a pressure of 1.01325 bar. A Nose–Hoover thermostat and a Martyna–Tobias–Klein barostat were used. Preliminary relaxation of the system was carried out for 2 ns. The simulation time was 300 ns, the number of frames was 5000, and the integrator step was 2 fs. Clustering of trajectories of the simulated protein-ligand complex was performed to find the most frequently occurring positions of the ligands in the active site of 3CL^pro^. The protein-ligand complexes’ free energy obtained as a result of the clustering was estimated by the MM-GBSA [44] method to identify the most energetically favorable configuration.

## 3. Results and Discussion

### 3.1. Synthesis of Usnic Acid Derivatives

**Figure 1:**
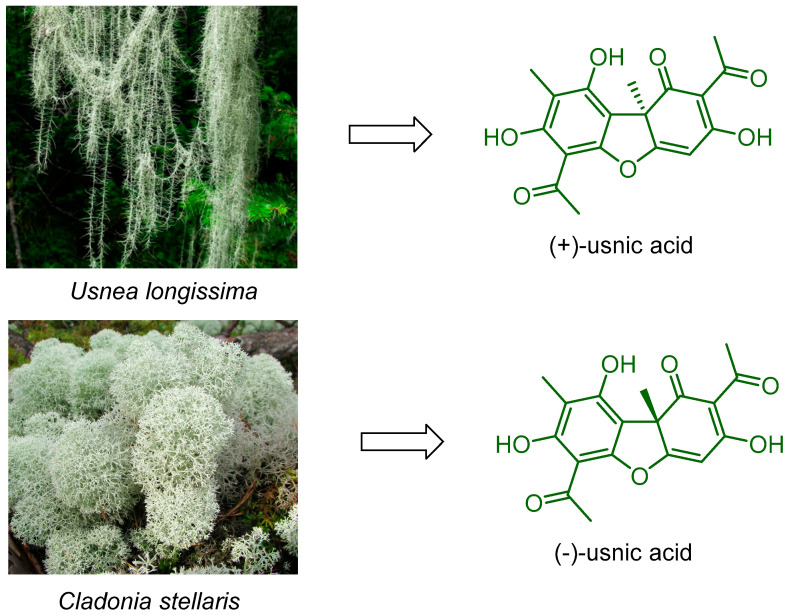
Structure of (+)-and (−)-usnic acid and their natural sources. The images are taken from [46] with permission.

Usnic acid is a secondary metabolite of the lichen genera *Usnea*, *Cladonia*, *Alectoria*, and many others. It has a wide range of biological activities: antimicrobial, antitumor, anti-inflammatory, and antiviral [45]. Usnic acid is produced in lichens in large quantities, accounting for up to 8% of thallus dry weight. Usnic acid, by its structure, belongs to the family of dibenzofuran derivatives and exists in two enantiomeric forms, differing in the configuration of the methyl group at the C_9b_ atom. *Usnea longissima* is a typical producer of (+)-usnic acid, whereas *Cladonia stellaris* can be considered a source of the levorotatory enantiomer of usnic acid (Figure 1). The widespread prevalence of various lichen species containing usnic acid, the simplicity of extraction from the raw materials, and the high purity of the isolated product make both enantiomers of this natural compound an excellent basis for the production of new pharmaceuticals.

Commercially available (+)-usnic acid **(+)-1** served as a starting compound. S-(−)-Usnic acid **(−)-1** was obtained by extraction from a mixture of the lichen *C. stellaris* and used as the starting material. 

**Scheme 1:**
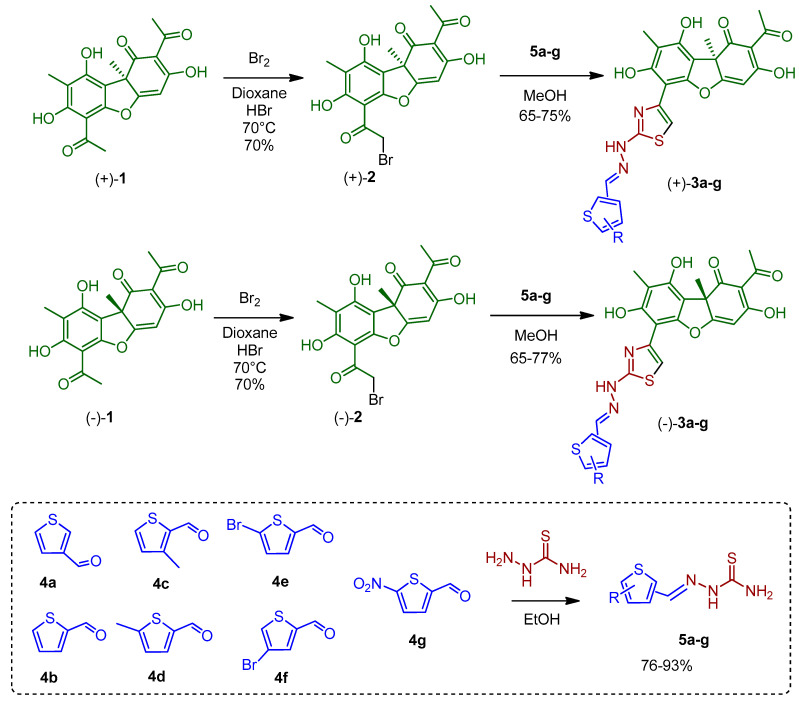
Synthesis of compounds **3a–g**.

Compounds **(+)-3a–g** and **(−)-3a–g** were synthesized by a method developed elsewhere [37]. Thiosemicarbazones **5a–g** were obtained through the reaction of corresponding thiophene aldehydes **4a–g** with thiosemicarbazide in ethanol with reflux (Scheme 1). The formed precipitate was filtered off, washed with water, and then air dried. Thiosemicarbazones **4a–g** were obtained with yields ranging from 76% to 93%. Bromo-substituted derivatives **(+)-2** and **(−)-2** were synthesized by a reaction of usnic acid with bromine in dioxane. Derivatives of usnic acid containing hydrazonothiazole moieties **(+)-3a–g** and **(−)-3a–g** were synthesized via the reaction of bromo-substituted derivatives **(+)-2** and **(−)-2** with thiosemicarbazones **5a–g** in methanol. The precipitate was filtered off, dissolved in methylene chloride, and washed with a sodium bicarbonate solution, and the organic phase was evaporated. Thus, usnic acid derivatives **(+)-3a–g** and **(−)-3a–g** were obtained with yields at the final step ranging from 65% to 78%.

As a result, we prepared a set of derivatives based on (+)- and (−)-usnic acid containing unsubstituted thiophenes or methyl-, bromo-, or nitrothiophenes. The structure of the obtained compounds was determined on the basis of ^1^H- and ^13^C-NMR spectra and high-resolution mass spectra.

### 3.2. Enzymatic Inhibition Assays with SARS-CoV-2 3CL^pro^

A number of studies involving virtual screening approaches [47,48,49] and our own data [25] indicate that the SARS-CoV-2 main protease can be a target for usnic-acid derivatives. Previously, we synthesized a set of thiazolo-hydrazones based on (+)- and (−)-usnic acid containing furan, pyrrole, indole, or p-methoxybenzylidene derivatives with different substituents at the *m*-position and investigated their ability to inhibit the main viral protease of SARS-CoV-2 [25]. Nine of 36 drugs were found to exhibit antiprotease activity in the range of 13 to 27 µM. Kinetic parameters of the four most active compounds were studied, and molecular modeling of the possible interaction of these compounds with the active site of the main protease was carried out [25]. At the same time, we noticed that the most active furan derivatives are not stable during storage. The thiophene derivatives of usnic acid synthesized in the present work are much more stable and do not degrade during storage. We investigated the antiprotease activity of the new usnic acid thiazole derivatives. The enzyme inhibition data are summarized in Table 1.

**Figure 2:**
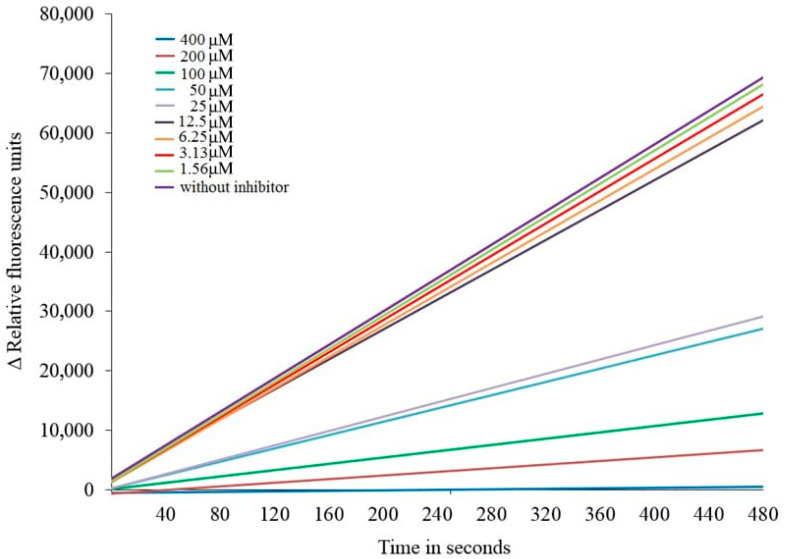
The curves of residual protease activity versus inhibitory concentration of compound **(+)-3e**.

We used nonselective (disulfiram and ebselen) and selective 3CL^pro^ inhibitors (ML188 and GC376) as reference compounds. All of the tested usnic-acid derivatives showed significantly lower activity than the reference agents did, and the activity of the latter were consistent with previously published data [50]. The results indicated that at 40–50 µM, the unsubstituted 2- and 3-thiophene derivatives of the (+) isomer of usnic acid, i.e., **(+)-3a,b**, and such derivatives of (−)-usnic acid, i.e., **(−)-3a,b**, possess moderate activity against 3CL^pro^. Compounds containing 2-methyl-substituted moieties, i.e., compounds **(+)-3d** and **(−)-3d**, also moderately inhibited the protease. Among the substances under study, a compound containing 2-bromo-substituted thiophene and synthesized from the (+)-isomer of natural usnic acid, namely **(+)-3e**, manifested the highest but not strong activity. Curves of residual protease activity depending on the inhibitor’s concentration are presented in Figure 2.

Its enantiomer **(−)-3e** inhibited the protease almost three times less effectively. Agents **(+)-3g** and **(−)-3g** (carrying 2-nitro thiophene-substituted moieties) showed no activity at all. 

### 3.3. Determination of the Kinetic Parameters of the Antiprotease Activity

**Figure 3:**
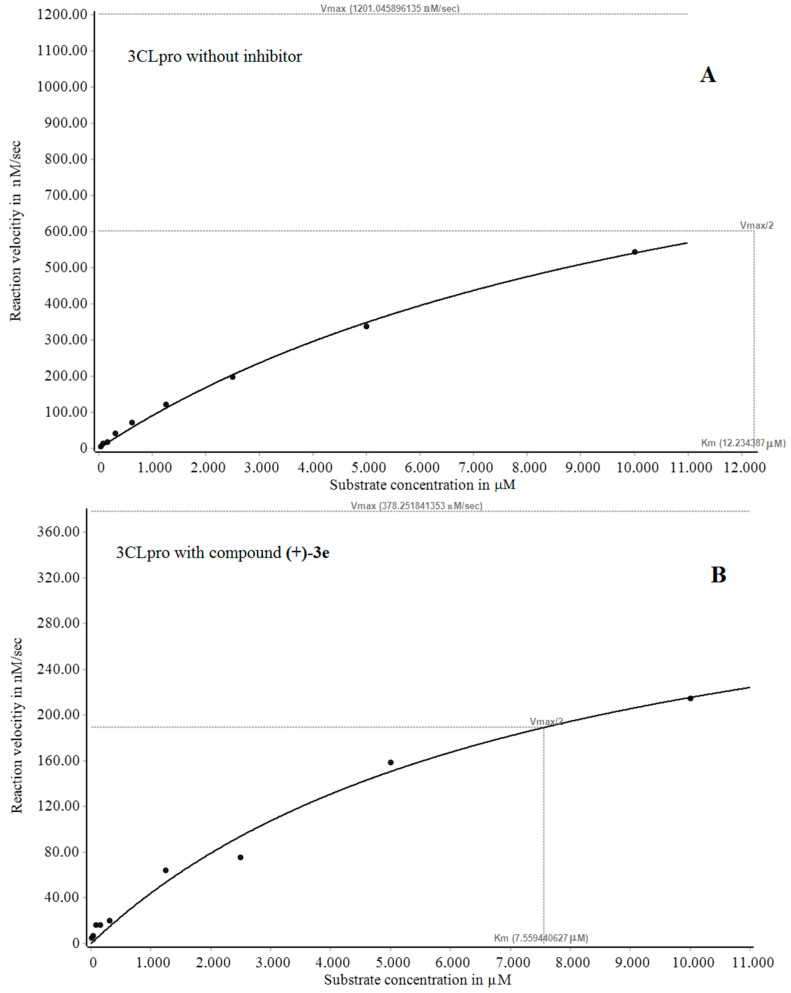
The dependence of the initial rate of substrate cleavage by SARS-CoV-2 3CL^pro^ without (**A**) or with a potential inhibitor **(+)-3e** (**B**). Each data point are an average of at least three independent experiments; the values are presented as the mean ± SE.

An important indicator of the biological action of a substance against an enzyme is its kinetic parameters. The rate of enzymatic reaction is determined by a change in the number of substrate or product molecules per unit time and reflects the catalytic activity of the enzyme. When a substance with an inhibitory effect is added to the reaction, the rate of enzymatic reaction may decrease due to the effect of the compound on the enzyme or enzyme-substrate complex. In the present work, we measured kinetic parameters for agents showing significant activity. To determine the kinetic parameters of the main coronavirus protease (with or without a potential inhibitor), fluorescence levels were measured at different substrate concentrations in kinetic scanning mode. The rate of enzymatic reaction was determined from kinetic fluorescence curves by calculation of a change in fluorescence per unit time at different substrate concentrations, and the results are summarized in Figure 3 and Table 2.

Our work revealed a significant decrease in the reaction rate in the presence of compound **(+)-3e** (as compared to the sample without the addition of an inhibitor) and a decrease in the Michaelis constant. The inhibition constant (K_i_), calculated as the K_m_/(K_m_^control^ − K_m_) ratio for inhibitor **(+)-3e** proved to be 1.43. A decrease in the Michaelis constant may indicate an increase in the affinity of the enzyme for the substrate; the reason is the unproductive formation of an inactive enzyme–substrate-inhibitor complex, and this process ultimately reduces the reaction rate.

### 3.4. Molecular Modeling

#### 3.4.1. Molecular Docking

Considering the 3CL^pro^-inhibitory activity that the new usnic-acid derivatives manifested, we performed molecular modeling of the possible interaction of these molecules with the active site of 3CL^pro^ to search for the most favorable conformations of these compounds in the active site and to identify possible noncovalent interactions of the atoms of the new derivatives with key catalytic amino acid residues of the enzyme, which is crucial for the replication cycle of the virus. As a result of the IFD protocol, parameter values corresponding to the best docking solutions were obtained; they are presented in Table 3.

As a result of the molecular docking, the most active M^pro^ inhibitor among the new compounds, **(+)-3e**, did not show high theoretical affinity for the active site of M^pro^. Therefore, the results of the molecular docking procedure do not explain the noticeable difference in the inhibitory ability of the compounds. This situation is not unique; many studies have yielded such results [51,52]. For this reason, a series of molecular dynamics simulations were carried out to assess the behavior of the ligands in the active site and the nature of their influence on the surrounding amino acid residues in 3CL^pro^. Ligand–protein complexes [of protease subunit 3CL^pro^ with either **(+)-3e** or **(+)-3g**] obtained as a result of the molecular docking procedure were selected for the molecular dynamics procedure. The choice was based on the results of the antiviral assay of these compounds: **(+)-3e** at 28.1 µM inhibited the viral protease, and **(+)-3g** did not show this activity. As a negative control, we examined the dynamics of the complex of subunit 3CL^pro^ with (+)-usnic acid **(+)-1**, which does not inhibit 3CL^pro^.

#### 3.4.2. Analysis of Molecular Dynamics Simulations

Examination of changes in the positions of ligand and protein atoms in the complexes under study led to the following conclusion: for the 3CL^pro^–**(+)-3e** and 3CL^pro^–**(+)-3g** systems, the simulation completed correctly, the range of changes in positions (root means square deviation; RMSD) of protein atoms did not exceed 2 Å in the last nanoseconds of simulations, and the position of ligands was stable relative to the position of the protein (Figures S1 and S2). For the 3CL^pro^–(+)-usnic acid complex, the ligand position was unstable throughout the simulation (Figure S3). Root mean square fluctuations (RMSFs) of the protein in complexes 3CL^pro^–**(+)-3e** and 3CL^pro^–**(+)-3g** were almost the same (Figure S4). The curve characterizing local shifts of atoms throughout the protein chain in the 3CL^pro^–(+)-usnic acid complex was noticeably different from the others. This finding suggests that the binding of (+)-usnic acid in the site is not stable and affects the side chains of surrounding amino acid residues.

**Figure 4:**
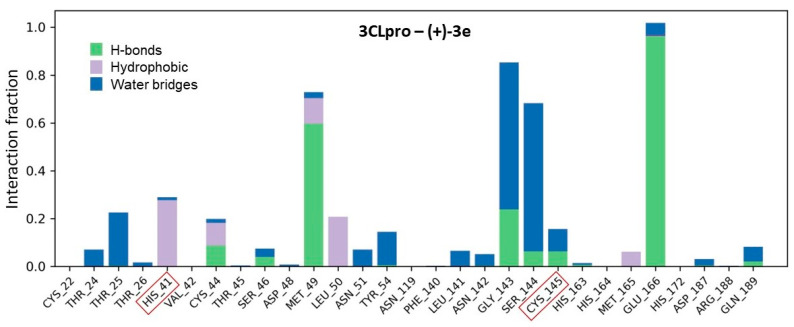
Contacts between atoms of the ligand and surrounding amino acid residues, as recorded throughout the entire simulation of the 3CL^pro^–**(+)-3e** complex. The amino acids of the catalytic dyad are highlighted with a red square.

Compounds **(+)-3e** and **(+)-3g** were found to contact approximately the same set of amino acid residues, including residues of the catalytic dyad. For instance, compound **(+)-3e** engaged in hydrophobic interactions with His41 for more than 25% of the simulation time and formed a hydrogen bridge with Cys145 for approximately 10% of the time (Figure 4). The longest-lasting hydrogen bridges involved Met49 and Glu166.

**Figure 5:**
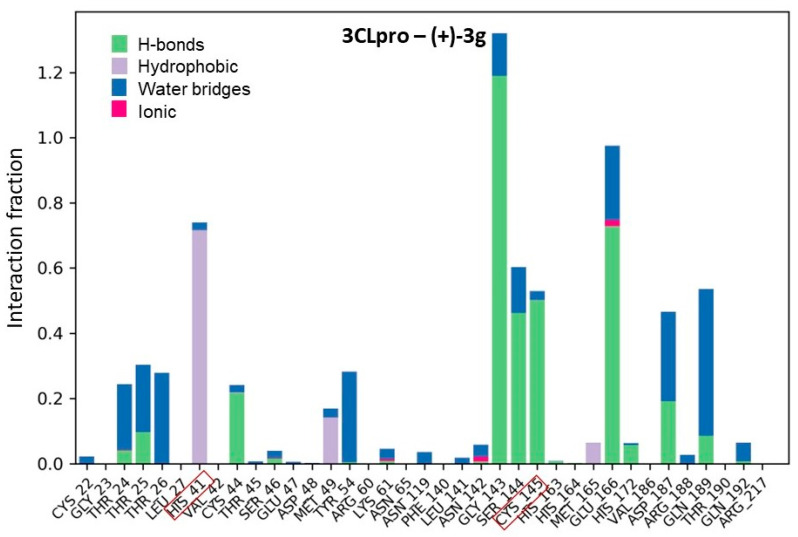
Contacts between atoms of the ligand and surrounding amino acid residues, as recorded throughout the entire simulation of the 3CL^pro^–**(+)-3g** complex. The amino acids of the catalytic dyad are highlighted with a red square.

The compound **(+)-3g** was found to contact a large number of amino acid residues. In this context, the hydrophobic contact with His41 was detectable for more than 70% of the total simulation time, and a hydrogen bridge with Cys145 was registered for >50%. A hydrogen bridge with Gly143 was detectable for more than 120% of the simulation time. This figure indicates that atoms of **(+)-3g** formed more than one hydrogen bridge with Gly143 throughout the simulation (Figure 5). Hydrogen bonding with Glu166 atoms was observed during 70% of the simulation time. In addition, for ~5% of the time, **(+)-3g** atoms engaged in ionic interactions (salt bridges) with Glu166 atoms.

During the entire simulation time, **(+)-1** was in contact with many amino acid residues, once again indicating an unstable position of the ligand in the binding site. Nevertheless, the molecule did not leave the binding site and did not diffuse into the solvent. The longest-lasting contacts included the hydrophobic contact with His41 and the hydrogen bridge with Glu166. For ~10% of the simulation time, hydrogen bridges with Cys145 were detectable (Figure S5).

**Figure 6:**
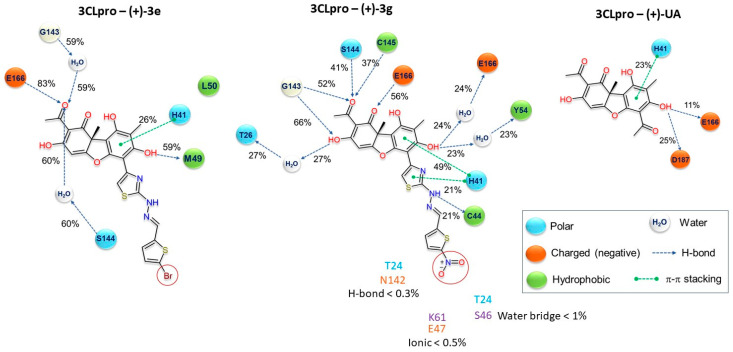
A summary of contacts recorded during molecular dynamics simulations. For complexes 3CL^pro^–**(+)-3e** and 3CL^pro^–**(+)-3g**, contacts are shown that were detectable for more than 20% of the simulation time for the 3CL^pro^–**(+)-1** complex, they were present for more than 10% of the simulation time. Various substituents are highlighted in red.

Analysis of the trajectories of the molecular dynamics simulations from the perspective of emerging contacts between ligand atoms and amino acid residues allowed us to notice that the longest-lasting contact is π-π stacking (hydrophobic contact) between the aromatic ring of the ligand and catalytic His41. Moreover, between **(+)-3g** and His41, this contact was detectable for the longest time as compared to contacts between His41 and **(+)-3e** and **(+)-1** (Figure 6). 

Furthermore, during 37% of the simulation time, a hydrogen bridge between carbonyl oxygen **(+)-3g** and catalytic Cys145 persisted. Between atoms of **(+)-3e**, **(+)-1**, and Cys145, hydrogen bonds were either not detectable or their duration was negligible. It should be noted that the difference between compounds **(+)-3e** and **(+)-3g** is only in the substituent at the fifth position of the thiophene ring. Indeed, the electronic natures of bromine and the nitro group differ; the bromine atom is rather prone to hydrophobic contacts with aliphatic amino acid residues, whereas the nitro group can form hydrogen and salt bridges with charged amino acid residues. Careful analysis of the trajectory of molecular dynamics simulations suggested that the bromine atom does not form contacts with surrounding amino acid residues, while short intermolecular interactions were noted between nitro atoms and surrounding amino acid residues (Figure 6).

Thus, the comparison of the nature of the behavior of the ligands **(+)-3e** and **(+)-3g** does not explain the reason for such different inhibitory activities. In this regard, the free energy of association of a ligand and protein into a ligand–protein complex was estimated. To this end, a clustering procedure was carried out, and the most statistically significant complex was determined, followed by the calculation of binding energy ΔG_bind_ by the MM-GBSA method (Table 4).

**Figure 7:**
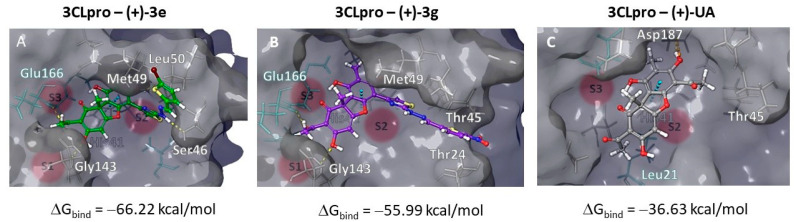
Molecular modelling study: Positioning of the studied ligands **(+)-3e** (**A**), **(+)-3g** (**B**) and **(+)-UA** (**C**), respectively, in the active binding site of subunit 3CLpro of the main protease. Letter designations S1–S3 correspond to significant cavities in the catalytic binding site.

Clustering of the results of molecular dynamics simulations enabled us to identify the most statistically significant ligand conformations, as presented in Figure 6. The usnic core of the compounds is located in the central part of the active site of 3CL^pro^ near the S2 subpocket [53] (Figure 7), with the formation of π-π stacking interactions with His41 (Table 4, Figure 7). The aceto group of the usnic core of **(+)-3e** and **(+)-3g** is located between subpockets S1 and S3. The binding of **(+)-3e** and **(+)-3g** differs in the location of the substituent: the bromine-containing substituent of compound **(+)-3e** is located closer to the hydrophobic amino acid Leu50 (Figure 7A), and the substituent with the nitro group of compound **(+)-3g** is situated between the residues of polar threonines at positions 24 and 45 (Figure 7B). No steric hindrance was observed in the positioning of **(+)-3e** and **(+)-3g**, in contrast to **(+)-1**, one of whose hydroxyl groups showed a clash with Asp187 (Figure 7C).

The results of the examination of molecular dynamics simulations led to the following conclusion: the positioning of a molecule of **(+)-3e** or **(+)-3g** in the active site of the protease subunit is stable. The binding energies differ between the two complexes; the active compound **(+)-3e** binds with minimal free energy, whereas the binding of the weakly active compound **(+)-3g** is characterized by higher values of ΔG_bind_. The positioning of the inactive **(+)-1** in the active site is not stable and is accompanied by the formation of intermolecular contacts with a large number of amino acid residues in the 3CL^pro^ active site. The free energy of the **(+)-1**–3CL^pro^ complex is the highest among the three compounds analyzed. Thus, the results of the molecular dynamics simulations are in agreement with the experimental data.

### 3.5. Biological Testing for an Infectious Virus 

The rapid spread of coronavirus infection worldwide has facilitated the rapid evolution of the virus, resulting in the emergence of multiple genetic variants of SARS-CoV-2. Mutations in the viral genome can affect transmissibility, virulence, disease course, sensitivity of diagnostic tests, vaccination efficacy, and the efficacy of antiviral drugs [54]. One of the most common SARS-CoV-2 variants responsible for massive COVID-19 outbreaks worldwide from 2021 onward was the Delta VOC variant, owing to its increased infectivity and ability to cause reinfections [55]. Omicron VOC was identified in November 2021; it quickly replaced the previous Delta variant and became the dominant variant worldwide. The first known genetic lineage of Omicron variant VA.1 features more than 30 mutations in the spike protein [56]. To date, the omicron variant has completely supplanted all other coronavirus variants. The rapid accumulation of mutations in different combinations has given rise to a large number of omicron sublineages, some of which are dominant worldwide [57]. 

We analyzed the inhibitory activity of compound **(+)-3e** against SARS-CoV-2 using six strains deposited in the State Collection of Viral Infectious Agents and Rickettsioses at the State Research Center of Virology and Biotechnology VECTOR (affiliated with Rospotrebnadzor), which belong to different genetic lines (B.1.1, B.1.617.2, BA.1, BA.5.2, BQ.1.1, and XBB.1.5). Strain hCoV-19/Russia/Omsk202118_1707/2020 (GISAID ID: EPI_ISL_1242008) of genetic lineage B1.1 (Wuhan variant) was isolated in 2020; viruses of this genetic lineage were circulating at the beginning of the pandemic. Strain hCoV-19/Russia/PSK-2804/2021 (GISAID ID: EPI_ISL_7338814) was isolated in April 2021; it belongs to genetic lineage B.1.617.2 (Delta variant). Four Omicron variant strains were used: strain hCoV-19/Russia/Moscow171619-031221/2021 (GISAID ID: EPI_ISL_8920444) of sublineage BA.1, which was the first known Omicron variant sublineage; strain hCov-19/Russia/Moscow-49415/2022 (GISAID ID: EPI_ISL_16613436) of sublineage BA.5.2; strain hCoV-19/Russia/TOM-SRC-8663/2023 (GISAID ID: EPI_ISL_17730076) of subvariant BQ.1.1; and strain hCoV-19/Russia/SAK-SRC-8527/2023 (GISAID ID: EPI_ISL_17730074) of Omicron subvariant XBB.1.5. Since the beginning of 2022, BA.5 has become the dominant variant [58]. One of the most widespread sublineages of Omicron is strain BA.5.2 [59]. Another direct descendant of BA.5 is the BQ.1.1 line; strains of this genetic sublineage are highly infectious and can evade an immune response [60]. Omicron subvariant XBB.1.5 is a sublineage of the XBB variant, which has resulted from a recombination of two BA.2 sublineages (BJ.1 and BM.1.1.1.1). Subvariant XBB contains 14 mutations in addition to those found in BA.2. The rapid growth of the number of XBB subvariants and their large set of peak mutations resemble the situation during the appearance of the first Omicron variant, BA.1 [61].

The assays were performed in a BSL-3 laboratory at the State Research Center of Virology and Biotechnology, VECTOR. EC_50_, CC_50_, and the selectivity index (SI) were determined for the studied compounds. The results are given in Table 5. We used remdesivir as a reference drug.

Our work revealed that the promising thiophene-containing derivative of usnic acid—**(+)-3e**—is not very toxic to the tested cell line. This compound’s activities against the above-mentioned strains of SARS-CoV-2 were comparable; the greatest activity was exerted by the substance against Omicron BA.1, BA.5.2, and XBB 1.5 strains. Compound **(+)-3e** possesses activity against all these viruses that is approximately one order of magnitude weaker than that of the reference drug; at the same time, it is important that our agent is active against all the tested strains.

## 4. Conclusions

In the present work, we synthesized 14 thiophene-thiazole derivatives of (+)- and (−)-usnic acid and investigated their activity against the SARS-CoV-2 main protease. We found that at 40–50 µM, unsubstituted 2- and 3-thiophene derivatives of the (+) isomer of usnic acid, i.e., **(+)-3a,b**, and such derivatives of (−)-usnic acid, i.e., **(−)-3a,b**, have moderate activity against 3CL^pro^. Compounds containing 2-methyl-substituted fragments [**(+)-3d** and **(−)-3d**] also moderately inhibited the protease. Among the evaluated substances, compound **(+)-3e**, which contains a 2-bromo-substituted thiophene moiety and was synthesized from the (+)-isomer of natural usnic acid, manifested the highest but not strong activity. Its enantiomer **(−)-3e** inhibited the protease almost three times less effectively. Compounds **(+)-3g** and **(−)-3g**, which carry 2-nitro thiophene-substituted moieties, showed no activity at all. 

To confirm the inhibitory properties of substance **(+)-3e**, we studied the kinetic parameters of its antiprotease activity. We observed a significant decrease in the reaction rate in the presence of **(+)-3e** in comparison with the experiment without an inhibitor and calculated the inhibition constant. We performed molecular modeling of possible interactions of these molecules with the active site of 3CL^pro^ to search for the most favorable conformations of these compounds in the active site and to identify possible noncovalent interactions of atoms of the new derivatives with key catalytic residues of this enzyme, which is key for the virus replication cycle.

Because the bromo-substituted derivatives of **3g** were not active at all, unlike all the other substances under study, we next carried out analyses using molecular dynamics techniques. The results of a molecular docking procedure do not allow us to explain such a noticeable difference in the inhibitory ability between the two compounds [**(+)-3e** and **(+)-3g**]. For this reason, we performed a series of molecular dynamics simulations to assess the behavior of the ligands in the binding site and the nature of the effect on the surrounding amino acid residues. Compounds **(+)-3e** and **(+)-3g** contact approximately the same set of residues, among which are key amino acids of the catalytic dyad. Nonetheless, the comparison of the nature of **(+)-3e** and **(+)-3g** behavior does not explain the reason for such different inhibitory activities. We evaluated the binding energies of the ligands and proteins in the ligand–protein complex. For this purpose, we performed the clustering procedure and determined the most statistically significant complex and estimated binding energy, ΔG_bind_. Although the results of the molecular dynamics simulation analysis showed that the positioning of a **(+)-3e** or **(+)-3g** molecule in the catalytic site of the protease subunit is stable, the binding energies differ substantially. For instance, active compound **(+)-3e** binds with minimum free energy; the binding of inactive compound **(+)-3g** is characterized by higher values of ΔG_bind_, whereas the positioning of the initial (+)-usnic acid is not stable and is accompanied by the formation of intermolecular contacts with many amino acid residues of the active site. Thus, the molecular dynamics results are consistent with the experimental data.

To understand whether usnic-acid derivatives are active not only against the main viral protease, we conducted an experiment on six strains of infectious SARS-CoV-2 viruses and demonstrated that **(+)-3e** is moderately active. Although its activity is lower than that of reference compounds, we believe that these results are quite important and may allow us to continue the synthesis of new derivatives of usnic acid in order to obtain effective inhibitors of a wide range of SARS-CoV-2 viruses.

This study also indicates that minimal structural differences between compounds have a dramatic effect on their biological activity. On the other hand, an attempt to explain the influence of structural differences between compounds on their activity by molecular modeling methods is not always successful when a limited number of calculation tools are used. Only an integrated approach with a deep dive into the simulation of ligand–target interactions allows one to evaluate the differences in energy parameters that reflect the impact of structural differences between molecules on their activity.

## Figures and Tables

**Table 1:** SARS-CoV-2 3CL^pro^–inhibiting activity of **(+)-3a–g**, **(−)-3a–g**, **(+)-1**, and **(−)-1** in comparison with inhibitors ML188, GC376, disulfiram, and ebselen.

**comp**	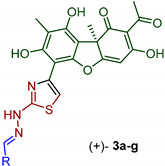	IC_50_, µM	**comp**	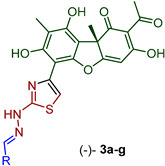	IC_50_, µM
**(+)-3a**	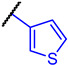	50.95 ± 6.33	**(−)-3a**	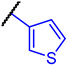	41.64 ± 4.97
**(+)-3b**	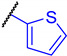	49.65 ± 6.12	**(−)-3b**	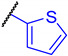	40.42 ± 5.65
**(+)-3c**	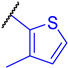	34.05 ± 4.45	**(−)-3c**	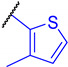	48.02 ± 6.21
**(+)-3d**	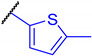	40.67 ± 5.11	**(−)-3d**	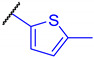	41.01 ± 5.89
**(+)-3e**	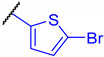	28.21 ± 1.41	**(−)-3e**	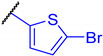	85.84 ± 10.15
**(+)-3f**	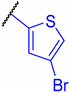	50.22 ± 6.75	**(−)-3f**	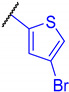	72.77 ± 9.55
**(+)-3g**	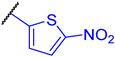	>200	**(−)-3g**	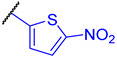	>200
**(+)-1**	–	>200	**(−)-1**	–	>200
**ML188**	–	1.56 ± 0.55	**Disulfiram**	–	6.25 ± 1.97
**GC376**	–	0.023 ± 0.004	**Ebselen**	–	1.72 ± 0.4

**Table 2:** Changes in the kinetic parameters of the enzymatic reaction with the addition of inhibitor **(+)-3e**.

Sample	IC_50_, µM	K_m,_ µM ^(a)^	V_max,_ nM/s ^(b)^	k_cat_, (s^−1^) ^(c)^	k_cat_/K_m_ (µM^−1^ s^−1^) ^(d)^
3CL^pro^	-	12.58 ± 1.99	1008.92 ± 96.15	3.36 ± 0.32	0.28 ± 0.05
**(+)-3e**	28.21 ± 1.41	7.40 ± 1.34	334.26 ± 39.08	1.11 ± 0.13	0.15 ± 0.01

^(a)^ the Michaelis constant: substrate concentration at which the reaction rate is half the maximum rate; ^(b)^ the maximum reaction rate; ^(c)^ the number of enzyme turnovers, i.e., the number of substrate molecules undergoing transformation in 1 min per active site or active enzyme molecule; ^(d)^ the catalytic efficiency of an enzyme is enzymatic activity at a constant number of molecules of that enzyme.

**Table 3:** Results of molecular docking of new usnic-acid derivatives into the active site of 3CL^pro^.

Ligand	Docking Parameters, kcal/mol (see Section 2.3.3)			
	Docking Score	LE	Emodel	IFD Score
**(−)-3f**	−14.403	−0.400	−118.365	−670.92
**(−)-3a**	−13.805	−0.394	−108.414	−668.88
**(+)-3c**	−13.336	−0.370	−142.033	−668.19
**(−)-3b**	−12.594	−0.360	−110.742	−667.56
**(+)-3a**	−12.089	−0.345	−117.862	−667.42
**(−)-3d**	−10.899	−0.303	−109.117	−665.55
**(−)-3c**	−10.739	−0.298	−111.977	−665.54
**(−)-3e**	−10.277	−0.285	−92.602	−665.06
**(+)-3b**	−10.689	−0.305	−93.545	−665.02
**(+)-3e**	−9.372	−0.260	−99.295	−664.68
**(+)-3f**	−8.376	−0.233	−85.940	−663.75
**(−)-3g**	−9.237	−0.243	−85.007	−663.54
**(+)-1**	−9.566	−0.383	−78.894	−662.44
**(−)-1**	−9.464	−0.379	−60.914	−661.46
**(+)-3d**	−7.295	−0.203	−74.813	−660.77
**(+)-3g**	−7.723	−0.203	−87.327	−660.67

**Table 4:** Free energies of complexes of ligands with M^pro^ and types of their noncovalent interactions with amino acid residues of the active site of M^pro^.

ID Complex	IC_50_ (3CL^PRO^), µM	ΔG_bind_, kcal/mol	Interaction with Amino Acids		
			HB	π-π Stacking	Others
3CL^PRO^–**(+)-3e**	28.21 ± 1.41	−66.22	Ser46 Met49 Gly143	His41	none
3CL^PRO^–**(+)-3g**	>200	−55.99	Gly143 Gly166	His41	none
3CL^PRO^–**(+)-1**	>200	−36.63	Asp187	His41	Asp187–clash

**Table 5:** Results from assays of antiviral activity of compound **(+)-3e** against SARS-CoV-2 viruses.

		Wuhan ^(a)^	Delta ^(b)^	Om. B.1. ^(c)^	Om. 5.2 ^(d)^	Om. XBB ^(e)^	Om. BQ ^(f)^
	CC_50_^(g)^	EC_50_^(h)^ (**SI**) ^(i)^	EC_50_ (**SI**)	EC_50_ (**SI**)	EC_50_ (**SI**)	EC_50_ (**SI**)	EC_50_ (**SI**)
**(+)-3e**	349.65 ± 21.2	25.3 ± 2.2 (**14**)	41.5 ± 3.1 (**8**)	20.7 ± 1.6 (**17**)	20.01 ± 1.6 (**17**)	22.4 ± 2.0 (**15**)	32.25 ± 2.6 (**11**)
Remdesivir	710.9 ± 21.2	3.8 ± 0.42 (**186**)	2.1 ± 0.16 (**338**)	2.0 ± 0.13 (**356**)	4.45 ± 1.0 (**159**)	2.65 ± 0.6 (**267**)	3.13 ± 0.9 (**226**)

^(a)^ Wuhan, lineages B.1.1: hCoV-19/Russia/Omsk202118_1707/2020 (GISAID ID: EPI_ISL_1242008); ^(b)^ Delta, lineage B.1.617.2: hCoV-19/Russia/PSK-2804/2021 (EPI_ISL_7338814); ^(c)^ Omicron, BA.1: hCoV-19/Russia/Moscow171619-031221/2021 (EPI_ISL_8920444); ^(d)^ Omicron, BA 5.2: hCov-19/Russia/Moscow-49415/2022 (EPI_ISL_16613436); ^(e)^ Omicron, XBB 1.5: hCoV-19/Russia/SAK-SRC-8527/2023 (EPI_ISL_17730074); ^(f)^ Omicron lineage BQ.1.1: hCoV-19/Russia/TOM-SRC-8663/2023 (EPI_ISL_17730076); ^(g)^ CC_50_ is half-maximal cytotoxic concentration, the concentration resulting in the death of 50% of cells, µM; ^(h)^ EC_50_ is half-maximal effective concentration, the concentration leading to 50% inhibition of virus replication, µM; ^(i)^ The selectivity index, i.e., the CC_50_/EC_50_ ratio. The presented data are the mean of three independent experiments. CC_50_ and EC_50_ are presented as the mean ± standard error.

## Data Availability

Raw data are available upon request.

## Supplementary Materials

The following supporting information can be downloaded at: https://www.mdpi.com/article/10.3390/v16020215/s1. Figures S1–S3: Fluctuation of root mean square deviation (RMSD) values of atoms in complexes 3CL^pro^–**(+)-3e**, 3CL^pro^–**(+)-3g**, and 3CL^pro^–**(+)-1**. Figure S4: Root mean square fluctuation (RMSF) of changes in the position of 3CL^pro^ atoms when a ligand is in the binding site. Figure S5: Contacts between atoms of the ligand and surrounding amino acid residues, as recorded throughout the entire simulation of the 3CL^pro^–**(+)-1** complex. Table S1: NMR ^1^H spectra of **3a–d;** Table S2: ^1^H-NMR spectra of **3e–g**; Table S3: ^13^C-NMR spectra of **3a–d**; Table S4: ^13^C-NMR spectra of **3e–g**; Figure S6–S19: ^1^H-NMR and ^13^C-NMR NMR pectra of compounds.
