# Supplementary material for: The Potential of Usnic-Acid-Based Thiazolo-Thiophenes as Inhibitors of the Main Protease of SARS-CoV-2 Viruses
Source: Viruses. 2024 Jan 31;16(2):215. doi: 10.3390/v16020215 (PMC10893357; doi:10.3390/v16020215)
Supplement: Supplementary file 1 [file viruses-16-00215-s001.zip › viruses-2767732-supplementary.pdf]

The Potential of Usnic-Acid-Based Thiazolo-Thiophenes as Inhibitors of the Main Protease of SARS-CoV-2 Viruses

Olga I. Yarovaya [1,*], Aleksandr S. Filimonov [1], Dmitriy S. Baev [1,2], Sophia S. Borisevich [2,3], Anna V. Zaykovskaya [4], Varvara Yu. Chirkova [5], Mariya K. Marenina [1], Yulia V. Meshkova [1], Svetlana V. Belenkaya [1,4], Dmitriy N. Shcherbakov [1,4,5], Maxim A. Gureev [6], Olga A. Luzina [1], Oleg V. Pyankov [4], Nariman F. Salakhutdinov [1] and Mikhail V. Khvostov [1]

[1] Department of Medicinal Chemistry, N.N. Vorozhtsov Novosibirsk Institute of Organic Chemistry SB RAS, 630090 Novosibirsk, Russia; alfil@nioch.nsc.ru (A.S.F.); mitja2001@gmail.com (D.S.B.); mareninamk@nioch.nsc.ru (M.K.M.); meshkova_29@mail.ru (Y.V.M.); belenkaya.sveta@gmail.com (S.V.B.); dnshcherbakov@gmail.com (D.N.S.); luzina@nioch.nsc.ru (O.A.L.); anvar@nioch.nsc.ru (N.F.S.); khvostov@nioch.nsc.ru (M.V.K.)

[2] Synchrotron Radiation Facility SKIF, G.K. Boreskov Institute of Catalysis SB RAS, 630559 Koltsovo, Russia; monrel@mail.ru

[3] Laboratory of Chemical Physics, Ufa Institute of Chemistry, Ufa Federal Research Centre, 450078 Ufa, Russia

[4] State Research Center of Virology and Biotechnology VECTOR, Rospotrebnadzor, 630559 Koltsovo, Novosibirsk Region, Russia; zaykovskaya_av@vector.nsc.ru (A.V.Z.); pyankov_ov@vector.nsc.ru (O.V.P.)

[5] Institute of Biology and Biotechnology, Altay State University, 656049 Barnaul, Russia; varvara.chirkova@gmail.com

[6] Laboratory of Bio- and Cheminformatics, St. Petersburg School of Physics, Mathematics and Computer Science, HSE University, 194100 St. Peterburg, Russia; max_technik@mail.ru

* Correspondence: ooo@nioch.nsc.ru

Supporting information

Analysis of molecular dynamics simulations

RMSD of atomic positions

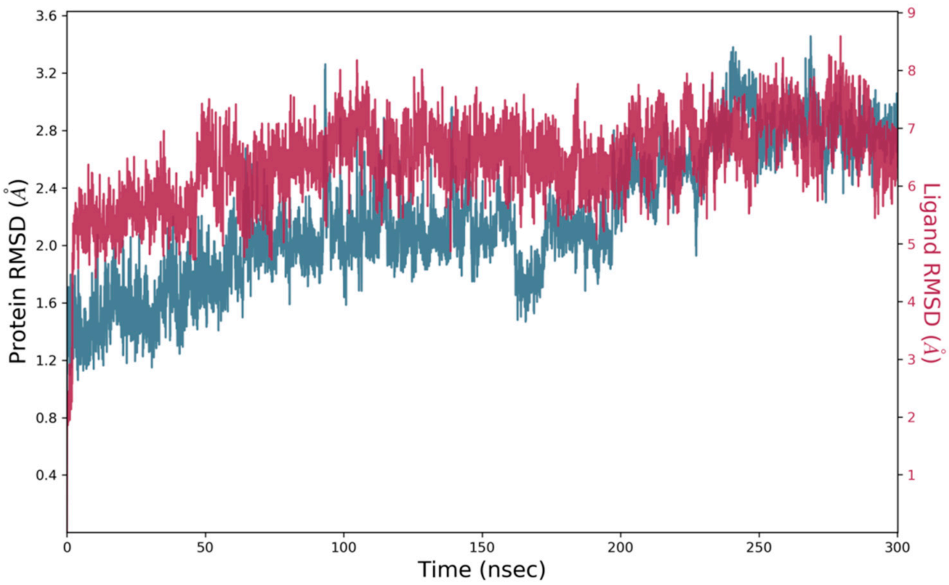

Figure S1. Fluctuation of the root mean square deviation (RMSD) values of atoms in the 3CLpro-**(+)-3e** complex.

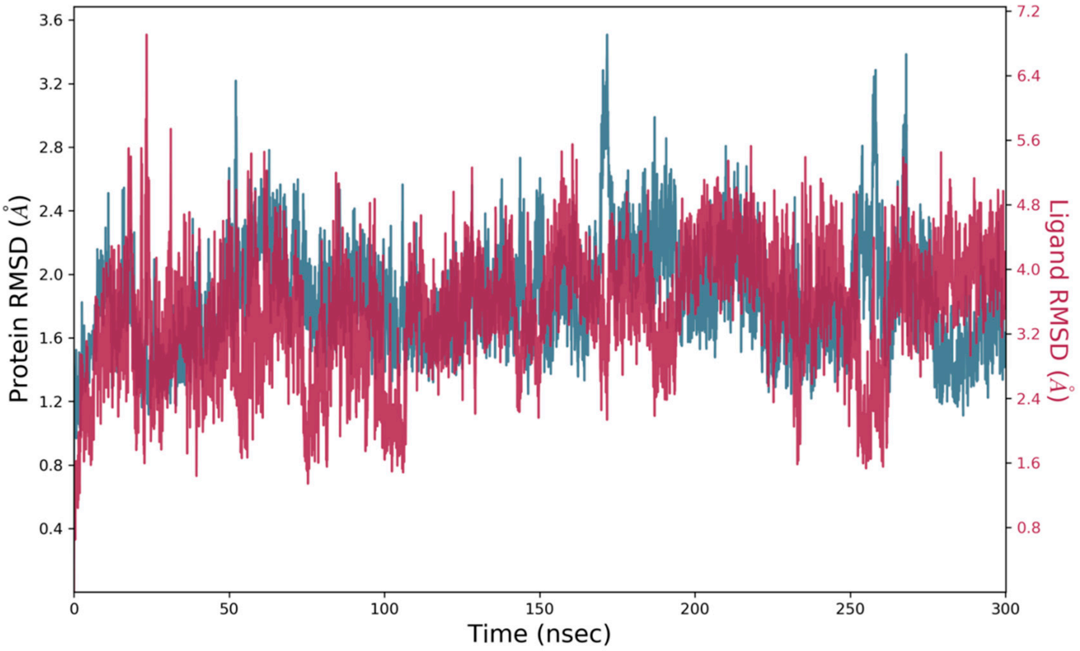

Figure S2. Fluctuation of the root mean square deviation (RMSD) values of atoms in the 3CLpro-**(+)-3g** complex.

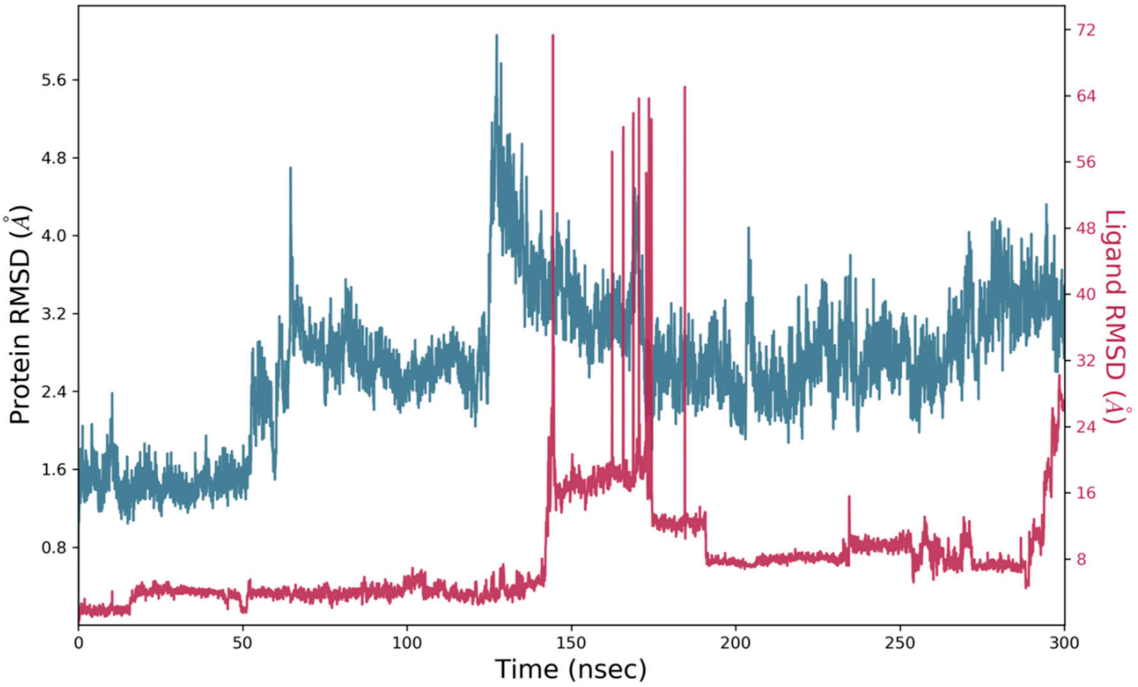

Figure S3. Fluctuation of the root mean square deviation (RMSD) values of atoms in the 3CLpro-**(+)-1** complex.

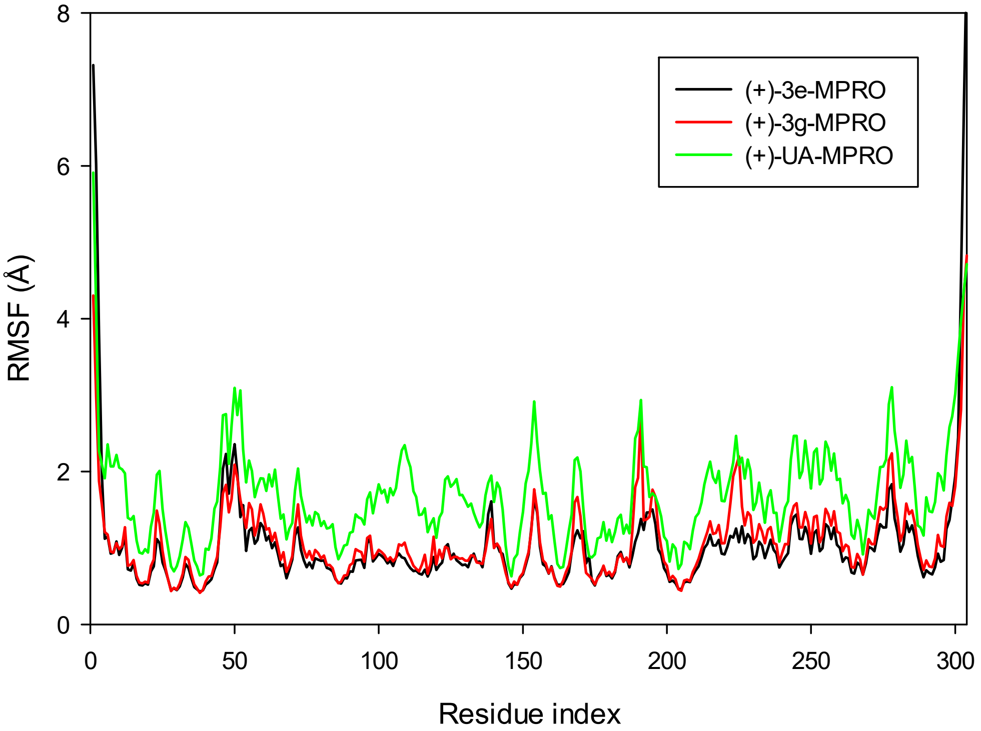

Figure S4. Root mean square fluctuation (RMSF) of changes in the position of MPRO subunit atoms with ligands in the binding site.

Registration of contacts between ligand and protein atoms

The figures below show histograms of registered contacts between ligand atoms and surrounding amino acid residues. 1.0 corresponds to 100%, i.e., one or another contact is recorded during the entire simulation time. Hydrogen bridges are detected at a distance of up to 2.5Å, at an angle of ≥120° in the combination D(donor)—H - - A(acceptor) or ≥90° in the combination H - - A(acceptor)—X.

Hydrophobic contacts include three types of interactions: π-π stacking, π-cation stacking, and other non-specific interactions, including contacts between hydrophobic amino acid residues and aromatic (and/or aliphatic) groups of the ligand. Contacts are recorded at a distance of 4.5 Å between aromatic and charged groups (π-cation), π-π sandwich or "T-form" stacking; nonspecific hydrophobic interactions up to 3.6Å.

Ionic interactions (or salt bridges) are detected at a distance of 3.4 Å between charged atoms. Water bridges correspond to contacts of the ligand with the atoms of amino acid residues through a water molecule. Bonds are recorded at a distance of up to 2.8 Å, at an angle of ≥110° in the combination D(donor)—H - - A(acceptor) or ≥90° in the combination H - - A(acceptor)—X.

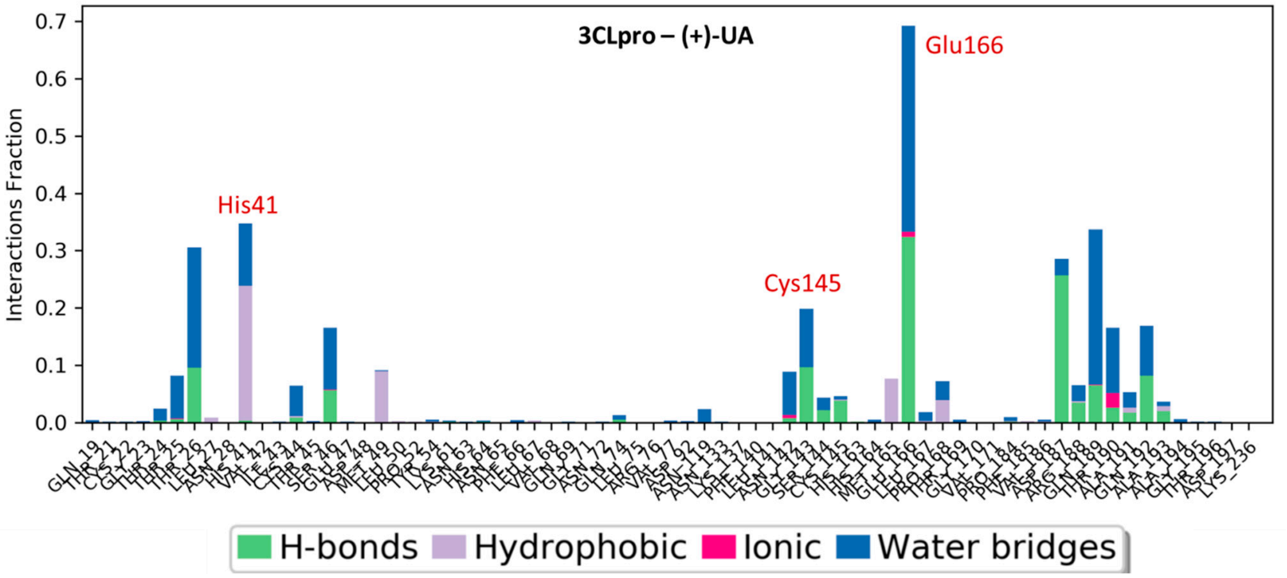

Figure S5. Contacts between atoms of the ligand and surrounding amino acid residues recorded throughout the entire simulation of the 3CLpro–**(+)-1** complex.

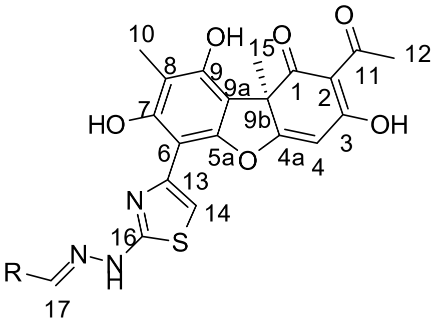

The ^{1}H and ^{13}C-NMR spectra of compounds 3a-f was recorded using $CDCl_3$ as a solvent. The ^{1}H and ^{13}C-NMR spectra of compounds 3g was recorded using $CDCl_3$ as a solvent.

Spectra NMR ^{1}H (**$CDCl_3$**, δ): 1.67 (3H, s, H-15), 2.13 (3H, s, H-10), 2.61 (3H, s, H-12), 5.88 (1H, s, H-4). Spectra NMR ^{13}C (**$CDCl_3$**, δ): 8.3 (C-10), 27.6 (C-12), 32.0 (C-15), 59.3 (C-9b), 97.2 (C-4), 97.3 (C-9a), 103.3 (C-6), 104.4 (C-14), 105.0 (C-2), 108.8 (C-8), 143.4 (C-13), 151.3 (C-7), 151.4 (C-9), 156.3 (C-5a), 166.4 (C-16), 180.4 (C-4a), 191.5 (C-3), 197.9 (C-1), 201.2 (C-11).

Spectra NMR ^{1}H (**DMSO-d_6**, δ): 1.70 (3H, s, H-15), 2.03 (3H, s, H-10), 2.60 (3H, s, H-12), 6.20 (1H, s, H-4). Spectra NMR ^{13}C (**DMSO-d_6**, δ): 8.3 (C-10), 27.6 (C-12), 32.0 (C-15), 59.0 (C-9b), 96.9 (C-9a), 97.3 (C-4), 103.3 (C-6), 105.1 (C-2), 105.6 (C-14), 107.3 (C-8), 143.0 (C-13), 151.3 (C-7), 151.4 (C-9), 156.3 (C-5a), 166.4 (C-16), 180.4 (C-4a), 191.5 (C-3), 197.9 (C-1), 201.2 (C-11).

Table S1: Spectra NMR ^{1}H of **3a-3d** ($CDCl_3$, δ):

№	3a	3b	3c	3d
**Structure**	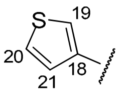	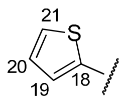	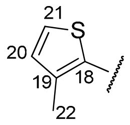	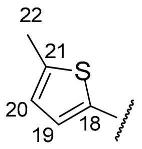
**H-14**	s 7.12	s 7.08	s 7.09	s 7.23
**H-17**	s 7.78	s 7.81	s 7.73	s 8.16
**H-19**	s 7.43	d 7.08 (J=3.4 Hz)		d 7.19 (J=4.9 Hz)
**H-20**	d 7.46 (J=4.9 Hz)	m 6.94	d 6.72 (J=5.3 Hz)	m 6.79
**H-21**	m 7.31	d 7.27 (J=5.0 Hz)	d 7.14 (J=4.9 Hz)	
**H-22**			s 2.23	s 2.46
**NH**	bs 8.99	bs 9.48	bs 9.11	---
**OH-3**	s 18.79	s 18.78	s 18.78	bs 18.82
**OH-7**	---	---	---	s 12.27
**OH-9**	s 10.28	s 10.27	s 10.26	s 10.27

Table S2: Spectra NMR ^{1}H of **3e-3g** ($CDCl_3$, δ):

№	3e	3f	3g
**Structure**	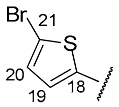	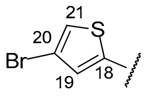	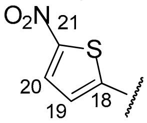
**H-14**	s 7.11	s 7.04	s 7.27
**H-17**	s 7.58	bs 8.12	s 8.08
**H-19**	d 6.77 (J=3.8 Hz)	s 7.04	d 7.34 (J=4.5 Hz)
**H-20**	d 6.86 (J=3.8 Hz)		d 7.98 (J=4.5 Hz)
**H-21**		s 7.19	
**H-22**			
**NH**	bs 9.06	---	s 12.78
**OH-3**	s 18.79	s 18.79	bs 18.78
**OH-7**	---	---	s 12.49
**OH-9**	s 10.29	s 10.40	s 10.23

Table S3: Spectra NMR ^{13}C of **3a-3d** ($CDCl_3$, δ):

**№**	**3a**	**3b**	**3c**	**3d**
**Structure**	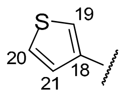	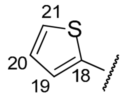	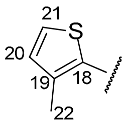	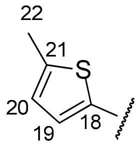
**C-17**	137.90	142.67	136.47	138.62
**C-18**	136.51	138.12	131.71	136.51
**C-19**	124.77	137.70	138.95	130.36
**C-20**	126.09	127.87	126.73	126.38
**C-21**	126.59	129.21	130.60	142.34
**C-22**			13.95	15.35

Table S4: Spectra NMR ^{13}C of **3e-3g** ($CDCl_3$, δ):

**№**	**3e**	**3f**	**3g**
**Structure**	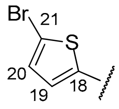	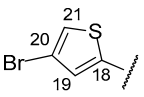	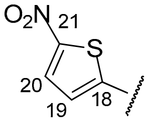
**C-17**	135.97	138.68	135.56
**C-18**	139.39	138.72	146.60
**C-19**	128.64	131.84	130.58
**C-20**	129.92	110.30	128.13
**C-21**	115.29	125.35	149.84
**C-22**			

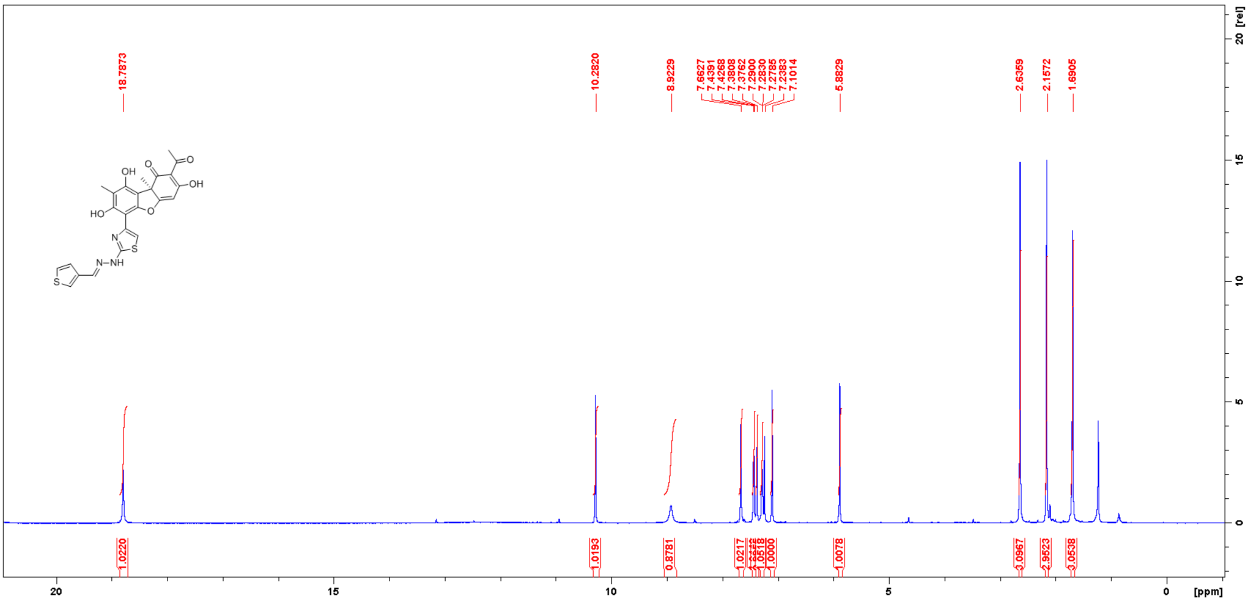

Figure S6. ^{1}H NMR Spectrum of compound (+)-3a

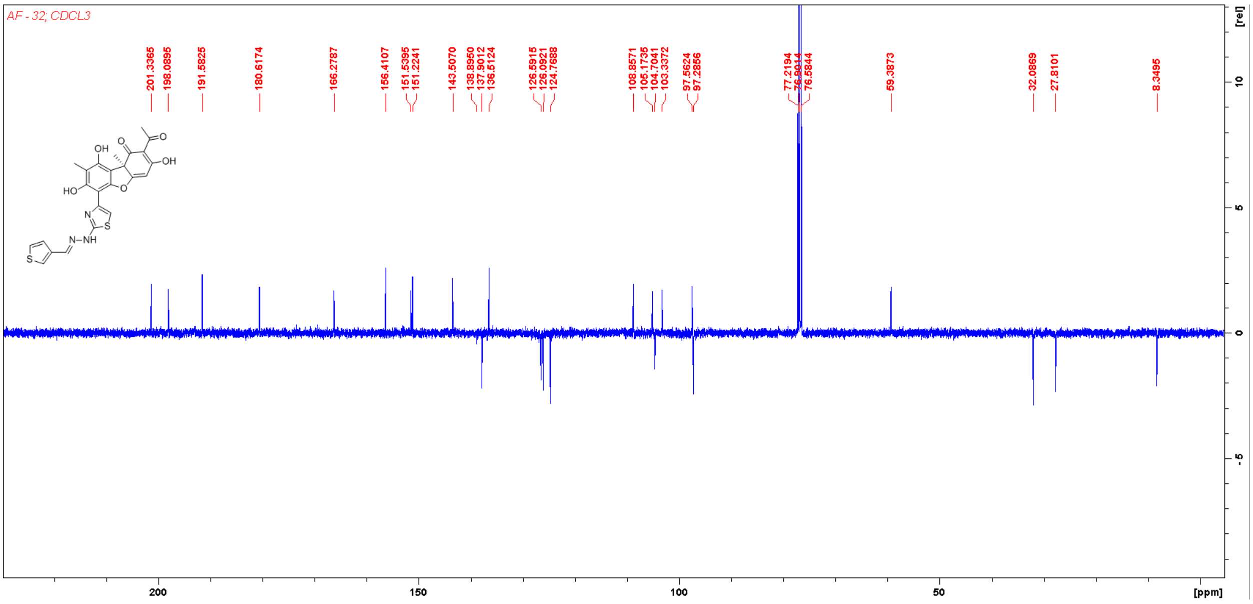

Figure S7. ^{13}C NMR Spectrum of compound (+)-3a

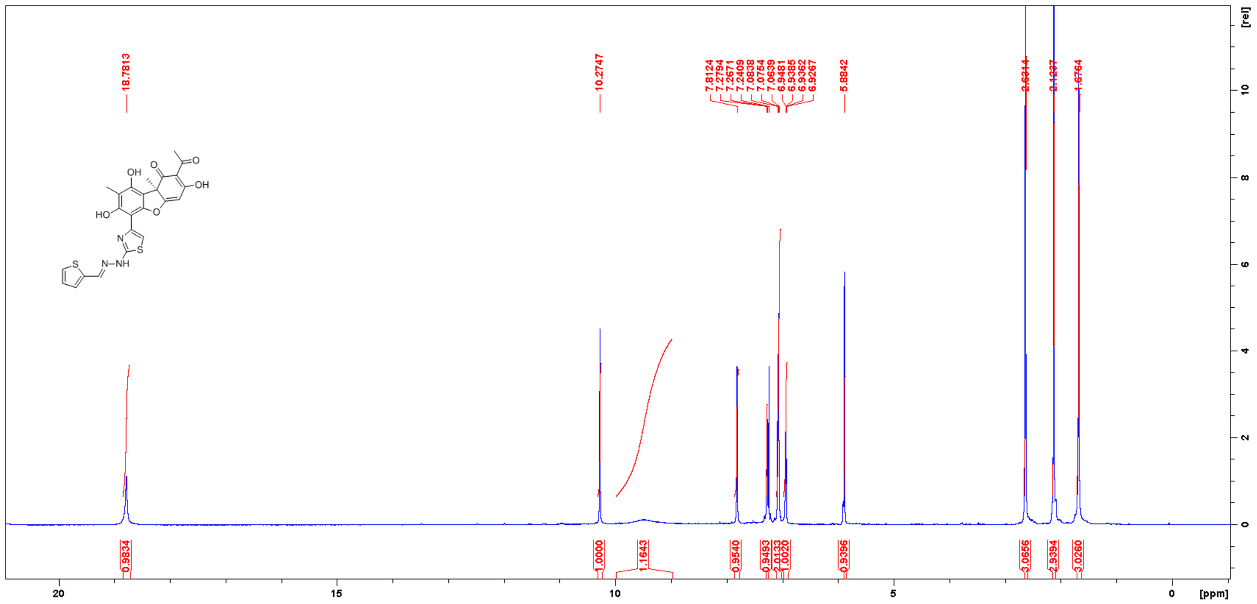

Figure S8. ^{1}H NMR Spectrum of compound (+)-3b

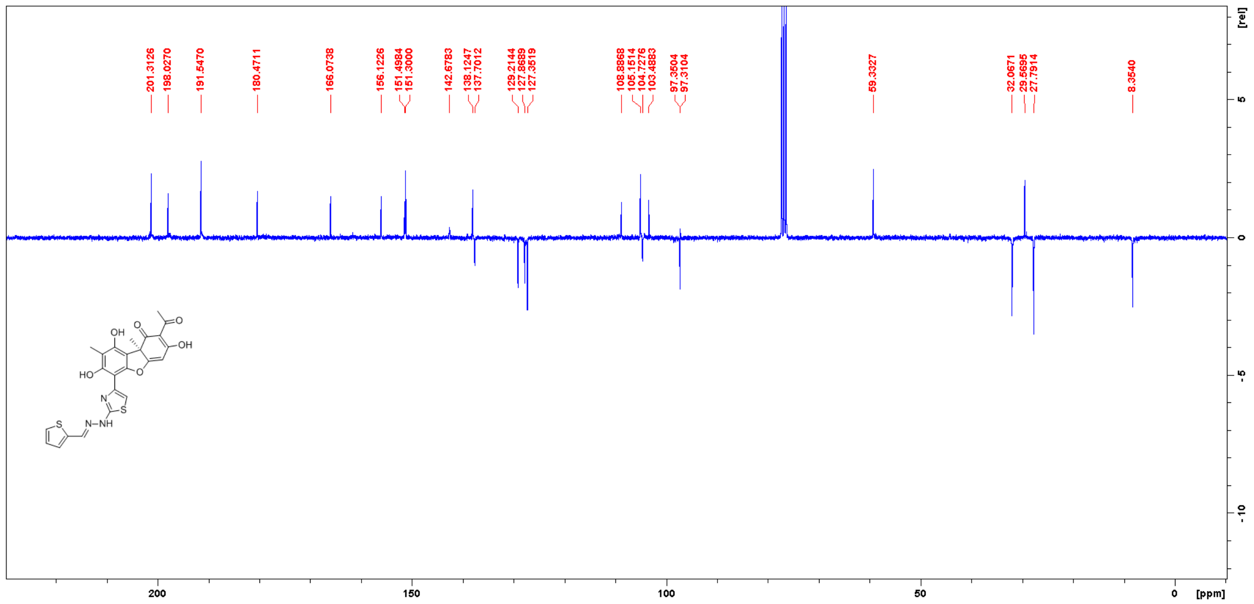

Figure S9. ^{13}C NMR Spectrum of compound (+)-3b

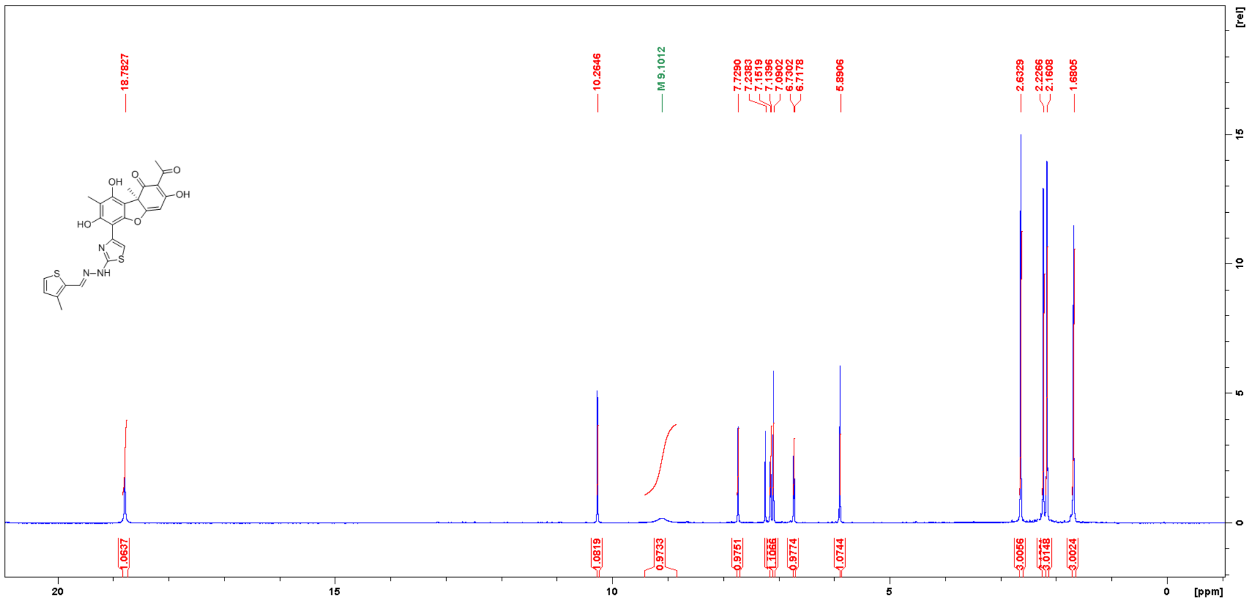

Figure S10. ^{1}H NMR Spectrum of compound (+)-3c

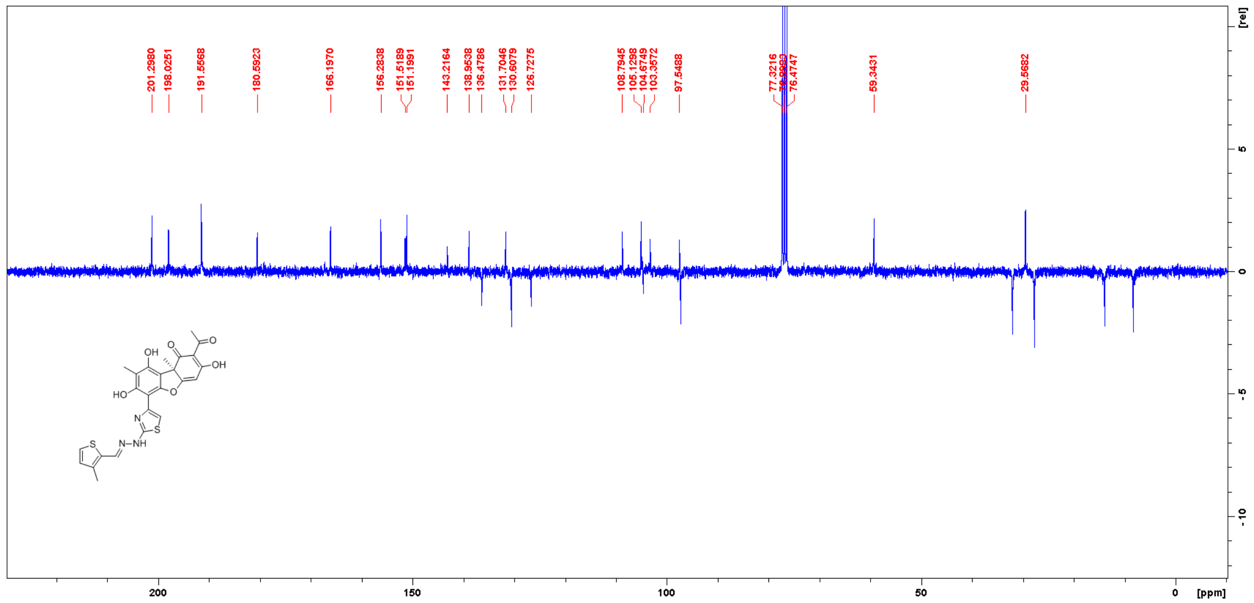

Figure S11. ^{13}C NMR Spectrum of compound (+)-3c

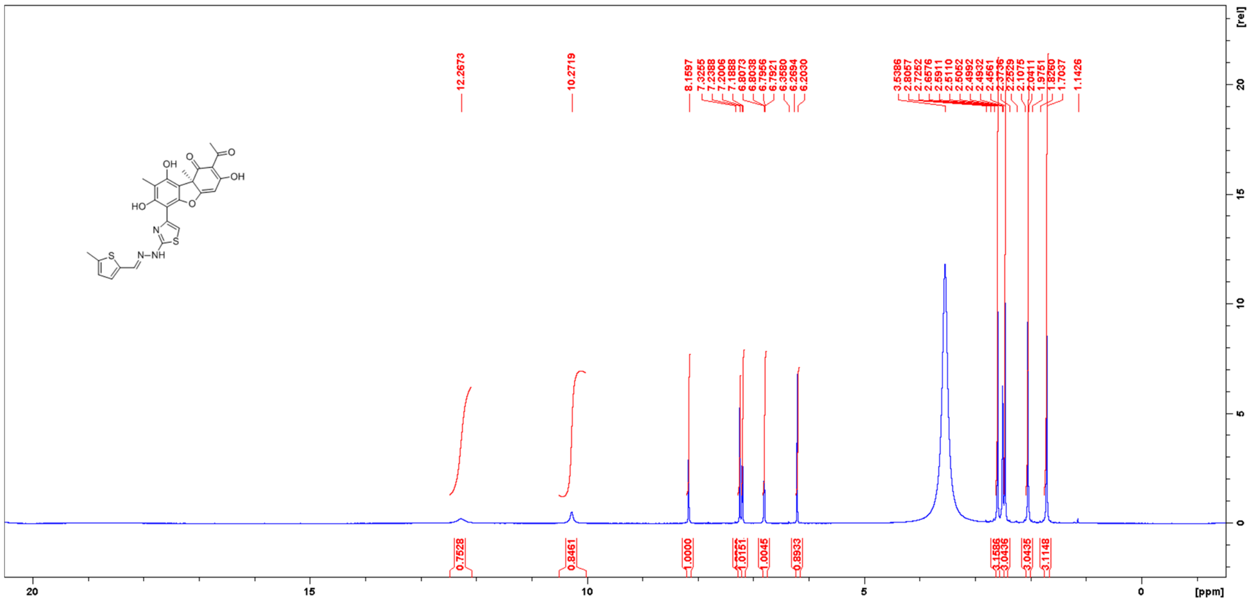

Figure S12. ^{1}H NMR Spectrum of compound (+)-3d

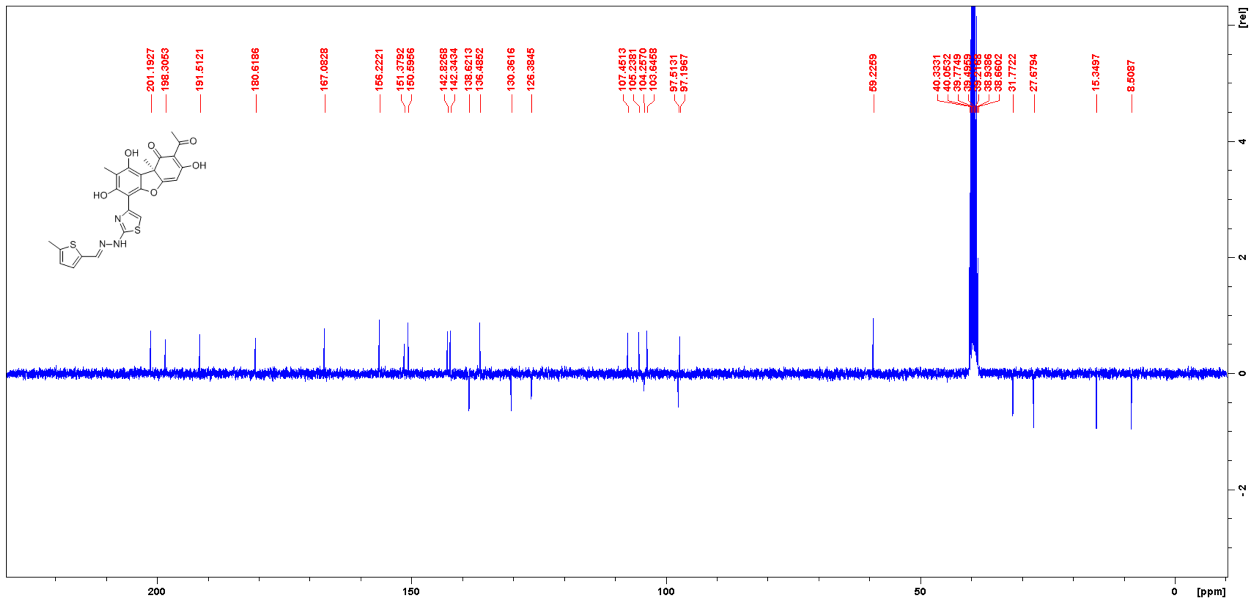

Figure S13. ^{13}C NMR Spectrum of compound (+)-3d

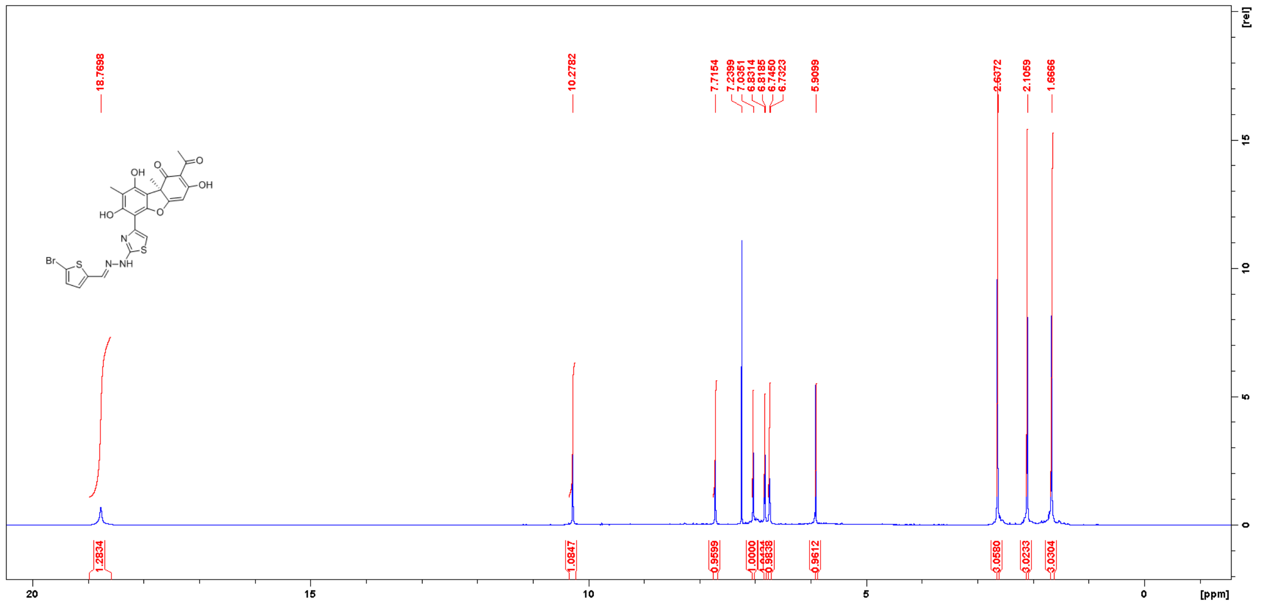

Figure S14. ^{1}H NMR Spectrum of compound (+)-3e

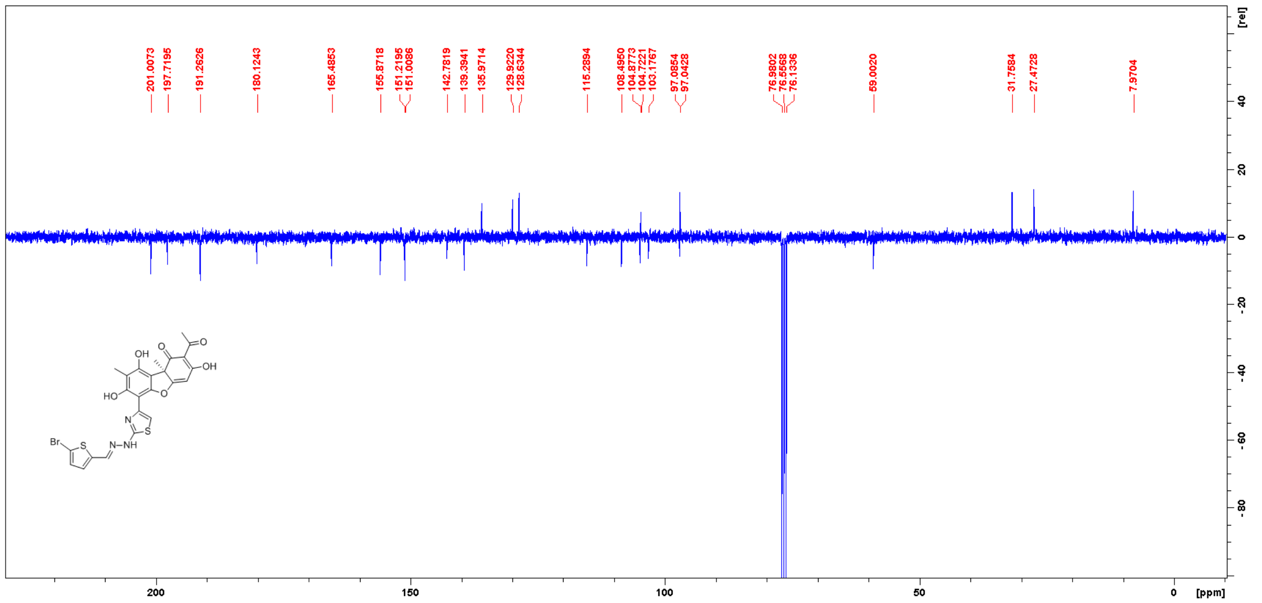

Figure S15. ^{13}C NMR Spectrum of compound (+)-3e

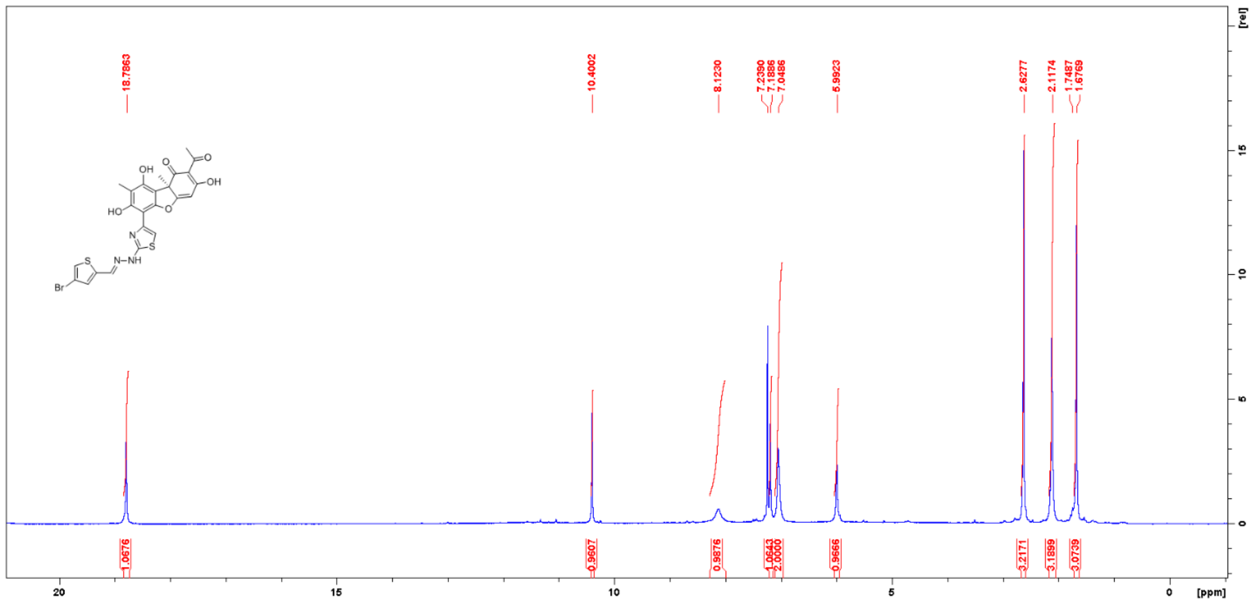

Figure S16. ^{1}H NMR Spectrum of compound (+)-3f

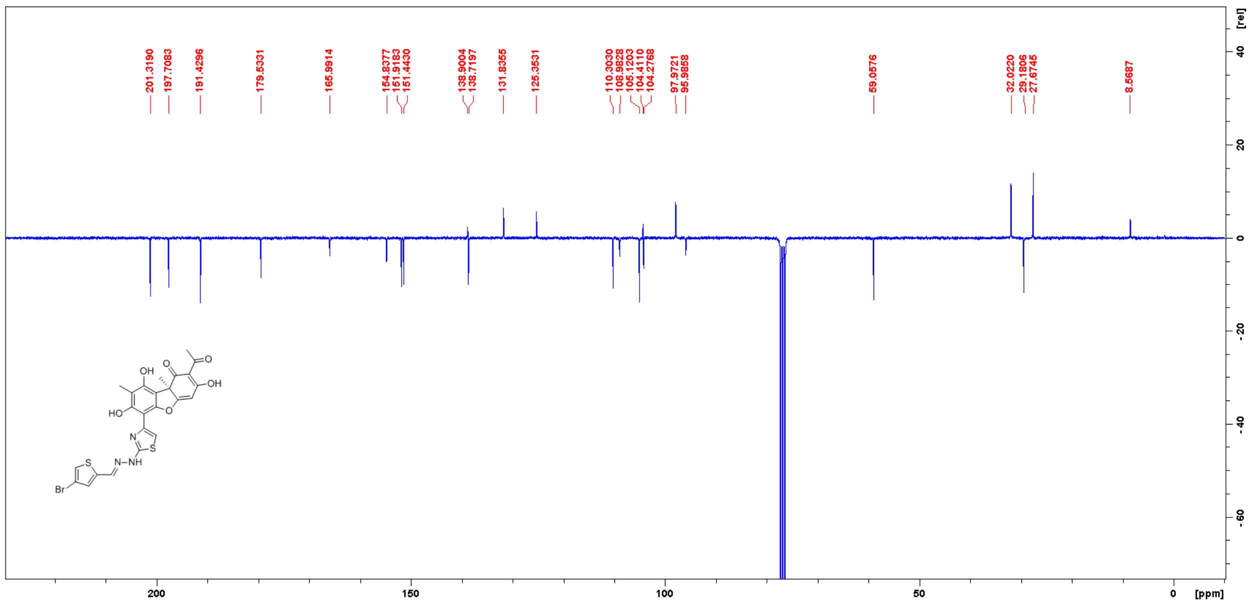

Figure S17. ^{13}C NMR Spectrum of compound (+)-3f

7

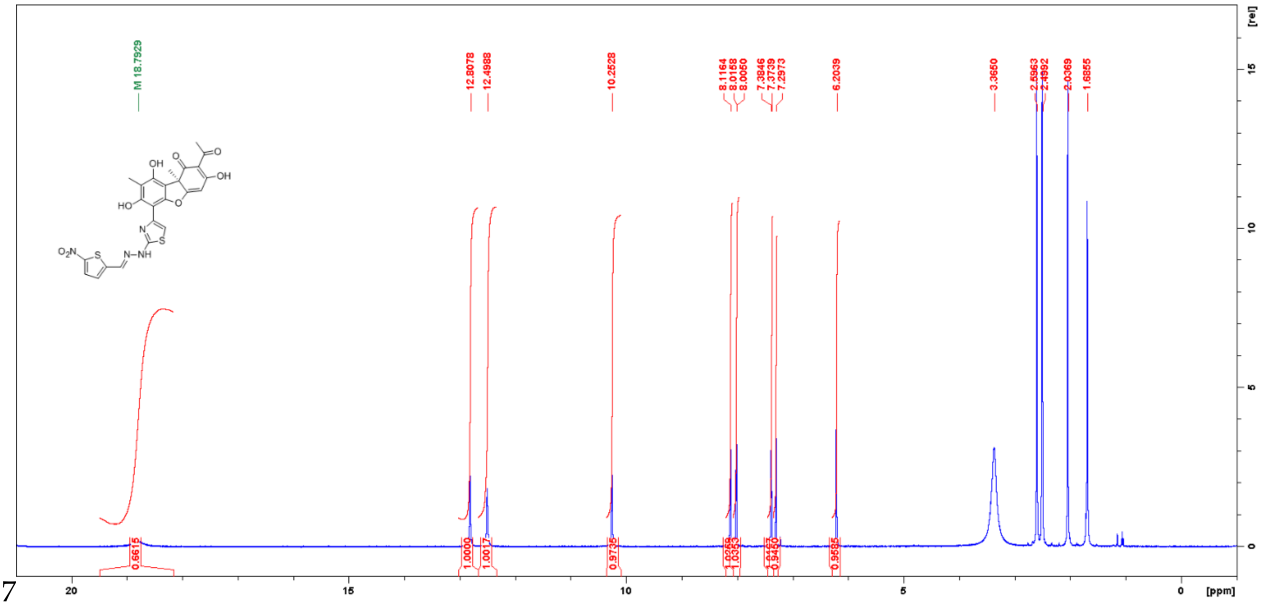

Figure S18. ^{1}H NMR Spectrum of compound (+)-3g

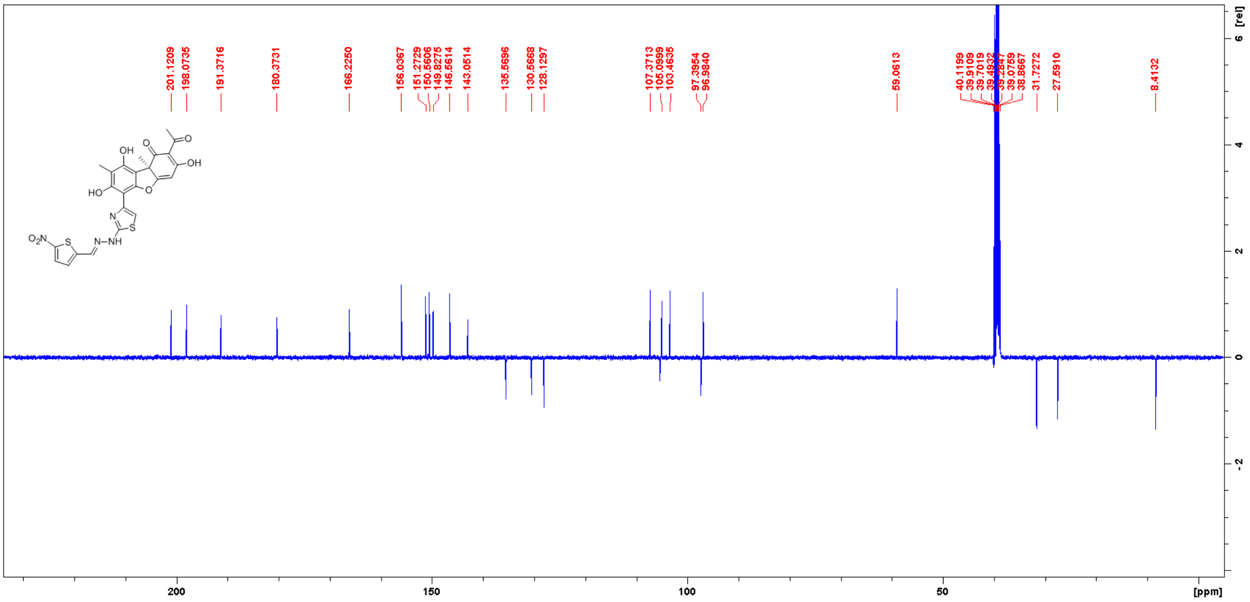

Figure S19. ^{13}C NMR Spectrum of compound (+)-3g
